# Chrono-Nutritional Disruption as a Potential Modifiable Factor in Bipolar Disorder

**DOI:** 10.3390/nu18183011

**Published:** 2026-09-14

**Authors:** Marianna Mazza, Giuseppe Marano

**Affiliations:** 1 Department of Neurosciences, Fondazione Policlinico Universitario Agostino Gemelli IRCCS, Università Cattolica del Sacro Cuore, 00168 Rome, Italy; 2 Department of Neurosciences, Section of Psychiatry, Fondazione Policlinico Universitario Agostino Gemelli IRCCS, 00168 Rome, Italy

**Keywords:** bipolar disorder, chrono-nutrition, circadian rhythms, feeding–fasting cycles, metabolic flexibility, clock genes, insulin resistance, gut microbiota, time-restricted eating, mood stabilization

## Abstract

Bipolar disorder is characterized by marked circadian and metabolic vulnerability, but direct evidence linking the timing of food intake to its course remains sparse. This structured narrative review examines chrono-nutritional disruption, including irregular meal timing, nocturnal eating, prolonged or inconsistent fasting, eating-window variability, and eating jet lag, as a potential behavioral correlate and mechanistic contributor to bipolar instability. PubMed/MEDLINE, Scopus, and Web of Science were searched for literature published from January 2000 to 30 June 2026, supplemented by predefined foundational studies. Evidence was organized as direct bipolar-specific, indirect human, or translational/preclinical. The strongest direct findings are cross-sectional associations involving night eating, eveningness, sleep disturbance, and functioning; bipolar-specific longitudinal and completed interventional evidence remains insufficient. Indirect human and experimental studies suggest that biologically mistimed eating can alter peripheral-clock entrainment, glucose–insulin regulation, cortisol rhythmicity, mitochondrial function, inflammatory signaling, and gut microbial rhythms. On this basis, we propose a hypothesis-generating chrono-metabolic model in which food timing may interact bidirectionally with sleep, mood state, medication effects, and metabolic health. Routine attention to nutritionally adequate and feasible meal regularity may be considered within broader monitoring of social rhythms, but it has not been shown independently to prevent relapse or improve mood outcomes. Time-restricted eating is investigational in bipolar disorder and should be evaluated only in carefully monitored research or specialist contexts. Prospective within-person studies and adequately powered trials are required before chrono-nutritional strategies can be regarded as evidence-based bipolar interventions.

## 1. Introduction

Bipolar disorder is a severe and recurrent psychiatric condition characterized by episodes of depression, mania, or hypomania, frequently accompanied by persistent functional impairment, cognitive difficulties, reduced quality of life, and an increased risk of premature mortality [1]. Although its clinical definition is based primarily on episodic changes in mood, energy, and activity, bipolar disorder is increasingly understood as a systemic condition involving disturbances in sleep, circadian regulation, energy metabolism, immune-inflammatory signaling, and neuroendocrine function [1,2]. This broader conceptualization is clinically relevant because biological rhythm disruption and metabolic abnormalities are not merely secondary complications of the disorder but may interact with its onset, course, and treatment response [3,4].

Sleep and circadian disturbances are among the most consistent biological features of bipolar disorder and can be observed during depressive, manic, mixed, and euthymic phases [4,5]. Mania and hypomania are typically associated with a reduced need for sleep, delayed or irregular sleep timing, and increased nighttime activity, whereas bipolar depression may involve insomnia, hypersomnia, delayed sleep phase, or marked variability in sleep–wake schedules [4,6]. Importantly, abnormalities in sleep and daily rhythms may persist during clinical remission and have been associated with residual symptoms, impaired functioning, and a greater risk of mood episode recurrence [3,5]. Prospective evidence also suggests that low regularity of social rhythms may precede the onset of bipolar-spectrum episodes in vulnerable individuals, supporting the hypothesis that rhythm instability may represent both a trait-like vulnerability and a state-dependent marker of illness activity [7].

Circadian regulation is maintained through a hierarchically organized network of central and peripheral oscillators [8]. The suprachiasmatic nucleus of the hypothalamus acts as the principal central pacemaker and is predominantly synchronized by the environmental light–dark cycle, while clocks located in peripheral tissues, including the liver, pancreas, gastrointestinal tract, adipose tissue, skeletal muscle, and immune cells, are additionally responsive to behavioral and metabolic signals [8,9]. These oscillators regulate daily variation in sleep propensity, hormonal secretion, glucose tolerance, insulin sensitivity, lipid metabolism, body temperature, immune activity, and gastrointestinal function [9,10]. Synchrony among these systems allows physiological processes to occur at biologically appropriate times, whereas recurrent misalignment between central and peripheral clocks can impair metabolic homeostasis and increase vulnerability to cardiometabolic disease [11,12].

This circadian perspective is particularly relevant to bipolar disorder because metabolic dysregulation is highly prevalent in affected individuals [13,14]. Obesity, insulin resistance, dyslipidemia, type 2 diabetes, and metabolic syndrome contribute substantially to the excess medical morbidity and reduced life expectancy associated with the disorder [13,15]. Although psychotropic medications, reduced physical activity, smoking, socioeconomic adversity, and unhealthy dietary patterns contribute to this burden, they do not fully explain the close association between bipolar disorder and metabolic dysfunction [1]. Shared abnormalities involving mitochondrial bioenergetics, oxidative stress, inflammatory pathways, hypothalamic–pituitary–adrenal axis regulation, insulin signaling, and circadian clock function have therefore been proposed as mechanisms connecting affective and metabolic instability [2,16,17].

Nutritional psychiatry has traditionally investigated how dietary quality, specific nutrients, food groups, and overall dietary patterns may influence mental health [15,18,19]. In bipolar disorder, previous research has examined dietary inadequacy, obesity-promoting eating behaviors, omega-3 polyunsaturated fatty acids, antioxidant nutrients, Mediterranean-style dietary patterns, and the possible contribution of the gut–brain axis [20,21]. These approaches have substantially advanced understanding of the relationship between nutrition and mood disorders, but they have focused predominantly on what and how much individuals eat rather than when food is consumed [22]. Dietary composition and food timing should not, however, be considered interchangeable dimensions, because identical meals may produce different metabolic responses depending on their timing relative to the endogenous circadian phase [23,24].

Chrono-nutrition is an emerging field that examines the reciprocal relationships among the timing, regularity, frequency, and daily distribution of food intake, endogenous circadian rhythms, and metabolic health [22,25]. Its principal dimensions include the timing of the first and last daily caloric intake, the duration of the eating window, meal regularity, fasting duration, breakfast consumption, nocturnal eating, and the distribution of energy across the day [22,26]. Food intake acts as a particularly important temporal cue for peripheral oscillators, and changes in feeding schedules can shift metabolic rhythms even when the central light-entrained pacemaker remains relatively unchanged [27,28]. Consequently, eating at biologically inappropriate times may generate internal desynchronization between the central clock and peripheral metabolic tissues [24].

Experimental studies in humans indicate that circadian misalignment can impair glucose tolerance, reduce insulin sensitivity, alter energy expenditure, and modify appetite-regulating hormones independently of sleep duration and caloric intake [23,29]. Late food intake has also been associated with greater hunger, altered energy expenditure, and molecular changes in adipose tissue favoring energy storage [30]. Conversely, restricting food intake to a consistent daily interval may reinforce feeding-fasting rhythms and improve selected metabolic outcomes, although the magnitude and durability of these effects vary according to the population, eating window, intervention design, and baseline metabolic status [31,32]. These findings support the principle that food functions not only as a source of calories and nutrients but also as a temporal signal capable of influencing metabolic organization [22].

Several behavioral characteristics of bipolar disorder may increase susceptibility to chrono-nutritional disruption. Irregular sleep–wake schedules, evening chronotype, reduced daily routine, impulsivity, reward sensitivity, altered appetite, nocturnal activity, and medication-related changes in hunger may all interfere with the regular timing of food intake [4,33]. Mood episodes may themselves produce marked changes in eating behavior: manic states can be accompanied by reduced attention to meals, prolonged wakefulness, erratic caloric intake, or increased reward-driven consumption, whereas depressive episodes may involve delayed eating, meal skipping, emotional eating, or increased evening intake [17]. Chrono-nutritional disruption may therefore be both a consequence and a potential amplifier of affective instability, creating a bidirectional cycle in which mood symptoms destabilize feeding rhythms and disrupted feeding rhythms further impair circadian and metabolic regulation [17].

The concept of “metabolic jet lag” has recently been proposed to describe recurrent misalignment between behavioral rhythms, food intake, circadian timing, and energetic metabolism in bipolar disorder [17]. According to this model, individuals with bipolar disorder may have difficulty adapting energy production, substrate utilization, appetite, and activity to changing environmental and behavioral demands [17]. This framework is consistent with emerging chrono-metabolic accounts that link bipolar mood states to altered coordination between circadian timing and energy availability [34]. Direct evidence concerning meal timing in bipolar disorder remains limited, and most available knowledge has been extrapolated from studies of circadian biology, obesity, diabetes, shift work, sleep disruption, or other psychiatric populations [35].

The potential clinical relevance of chrono-nutrition in bipolar disorder is nevertheless beginning to attract attention. A recent clinical protocol has proposed time-restricted eating as an adjunctive intervention for individuals with bipolar disorder and sleep or circadian disturbances, with planned evaluation of mood symptoms, quality of life, dim-light melatonin onset, actigraphy, continuous glucose monitoring, clock-gene expression, and metabolic parameters [35]. The emergence of such studies indicates that the temporal organization of eating is moving from theoretical plausibility toward direct clinical investigation, while also emphasizing that efficacy and safety data remain preliminary [36].

The present narrative review therefore examines chrono-nutritional disruption as a potentially modifiable pathway linking circadian vulnerability, metabolic dysfunction, and mood instability in bipolar disorder. Rather than providing another general overview of dietary composition or nutritional supplementation, the review focuses specifically on the temporal organization of food intake, including meal timing, regularity, nocturnal eating, fasting intervals, daily caloric distribution, and alignment with individual chronotype. It integrates evidence from circadian biology, metabolic research, nutritional psychiatry, and bipolar disorder to clarify the mechanisms through which irregular feeding schedules may influence clock-gene regulation, glucose-insulin homeostasis, mitochondrial function, hypothalamic–pituitary–adrenal axis activity, immune-inflammatory signaling, and gut microbial rhythmicity. The review further proposes a hypothesis-generating, non-validated a bidirectional chrono-metabolic model in which mood episodes and chrono-nutritional disruption reinforce one another, critically evaluates the still-investigational role of structured meal timing and time-restricted eating, and identifies priorities for bipolar-specific longitudinal and interventional research.

## 2. Materials and Methods

### 2.1. Review Design, Search Strategy, and Scope

This study was conducted as a structured narrative review integrating clinical, translational, and mechanistic evidence on the temporal organization of food intake in bipolar disorder. A narrative design was selected because direct studies specifically addressing chrono-nutrition in bipolar disorder remain limited and the available evidence is heterogeneous in terms of populations, exposures, interventions, outcomes, and study designs. The review was developed in accordance with methodological principles for transparent and critical narrative reviews [37].

A literature search was conducted in PubMed/MEDLINE, Scopus, and Web of Science for peer-reviewed articles published from January 2000 to 30 June 2026. The year 2000 was selected a priori to capture contemporary clinical and molecular chronobiology while maintaining a manageable and clinically relevant evidence base. Earlier studies were eligible only when repeatedly cited as foundational demonstrations of food-entrainable oscillators, peripheral-clock regulation, or circadian effects of meal timing; these were identified by backward citation searching from recent reviews and by agreement of both authors. The reporting of the search strategy was informed by PRISMA-S recommendations, although the present work was not designed as a systematic review [38]. Complete database-specific strategies are provided in Supplementary Table S1.

Search terms covered four main domains: bipolar disorder and affective instability; circadian rhythms and chronotype; meal timing and chrono-nutrition; and metabolic, neuroendocrine, inflammatory, mitochondrial, and microbial mechanisms. The principal terms included “bipolar disorder”, “bipolar depression”, “mania”, “hypomania”, “mixed features”, “rapid cycling”, “mood instability”, “circadian rhythm”, “circadian disruption”, “circadian misalignment”, “chronotype”, “eveningness”, “social jet lag”, “social rhythm”, “chrono-nutrition”, “chrononutrition”, “meal timing”, “meal regularity”, “irregular eating”, “breakfast skipping”, “late eating”, “night eating”, “feeding–fasting cycle”, “eating window”, “time-restricted eating”, “time-restricted eating”, “intermittent fasting”, “caloric distribution”, “metabolic syndrome”, “insulin resistance”, “glucose variability”, “metabolic flexibility”, “mitochondrial dysfunction”, “cortisol”, “hypothalamic–pituitary–adrenal axis”, “inflammation”, “gut microbiota”, “microbial rhythms”, and “clock genes”.

Terms were combined using the Boolean operators AND and OR. A representative search string was:

(“bipolar disorder” OR “bipolar depression” OR mania OR hypomania OR “mixed features” OR “mood instability”) AND (“chrono-nutrition” OR chrononutrition OR “meal timing” OR “meal regularity” OR “late eating” OR “night eating” OR “feeding–fasting cycle” OR “time-restricted eating” OR “caloric distribution”) AND (“circadian rhythm” OR “circadian misalignment” OR chronotype OR “social jet lag” OR metabolism OR “insulin resistance” OR inflammation OR microbiota).

Additional searches combined bipolar disorder–related terms with specific mechanisms, including glucose–insulin regulation, mitochondrial bioenergetics, appetite-regulating hormones, hypothalamic–pituitary–adrenal axis function, inflammatory signaling, tryptophan metabolism, gut microbial rhythmicity, and clock-gene expression. Reference lists of relevant studies, reviews, meta-analyses, and consensus papers were also manually screened to identify additional publications.

### 2.2. Eligibility Criteria

Studies were eligible when they addressed at least one of the following areas: meal timing, meal regularity, nocturnal eating, breakfast consumption, fasting intervals, eating windows, or daily caloric distribution in bipolar disorder; relationships among eating behavior, chronotype, sleep–wake rhythms, metabolic regulation, and mood instability; metabolic, mitochondrial, neuroendocrine, inflammatory, genetic, or microbial pathways potentially linking food timing with affective regulation; chrono-nutritional or time-restricted eating interventions relevant to psychiatric, circadian, or metabolic outcomes; clinical factors capable of modifying eating timing in bipolar disorder, including mood episodes, psychotropic medications, impulsivity, reward sensitivity, and comorbid eating disturbances.

Eligible publications included randomized and non-randomized intervention studies, longitudinal and cross-sectional observational studies, cohort and case–control studies, systematic reviews, meta-analyses, consensus statements, and relevant translational or preclinical investigations.

Articles were excluded when they focused exclusively on dietary composition or nutritional supplementation without a temporal or circadian dimension; did not provide clinically or mechanistically relevant information for bipolar disorder; were conference abstracts, editorials, opinion papers, or non-peer-reviewed publications; were not available in English; or duplicated data reported in another publication. When several articles described the same cohort, the most complete report was prioritized, while companion publications were retained when they presented distinct outcomes. The English-language restriction may have introduced language bias.

### 2.3. Study Selection and Evidence Organization

**Figure 1:**
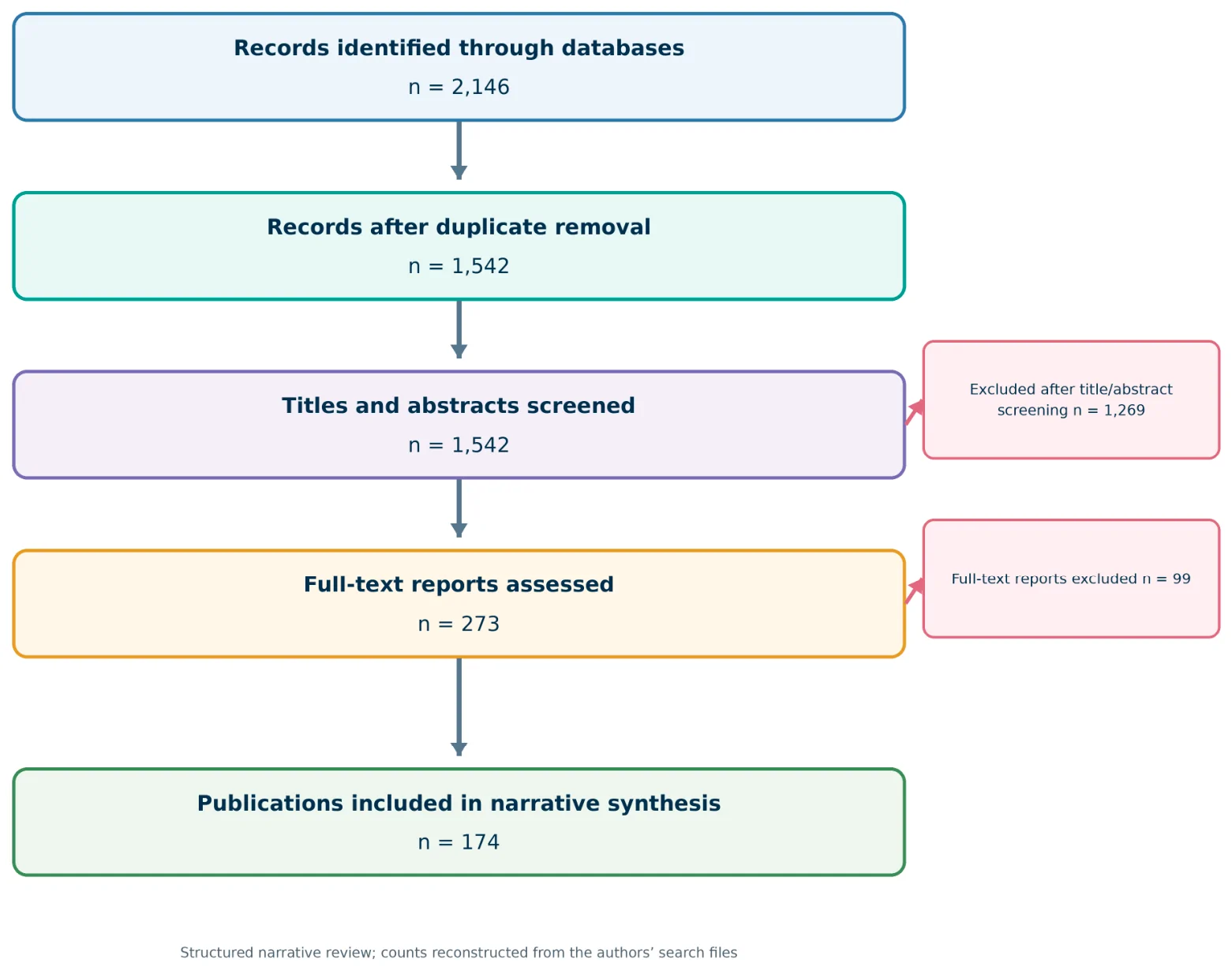
Flow of literature identification and selection for the structured narrative review. Counts were reconstructed from the authors’ search files during revision and do not represent a prospectively registered systematic-review process.

Both authors independently screened titles and abstracts, followed by independent full-text assessment of potentially eligible articles. Disagreements were resolved through discussion and consensus. The searches identified 2146 records; 1542 remained after duplicate removal and were screened, 273 full texts were assessed, and 174 publications were retained in the narrative evidence base. Because these counts were reconstructed from the authors’ search files for this revision, they should be interpreted as a transparent audit trail for a narrative review rather than as evidence of a prospectively registered systematic-review workflow (Figure 1). Priority was given to studies directly involving individuals with bipolar disorder, longitudinal and interventional designs, adequately characterized clinical samples, objective measures, and evidence not covered by previous reviews.

Because bipolar-specific chrono-nutritional evidence remains limited, the literature was organized into three hierarchical levels:

Direct evidence: studies conducted in individuals with bipolar disorder examining meal timing, eating regularity, night eating, chronotype-related eating patterns, metabolic rhythms, or chrono-nutritional interventions;

Indirect clinical evidence: studies conducted in other psychiatric disorders, sleep and circadian disorders, obesity, metabolic syndrome, or type 2 diabetes when their findings were considered relevant to bipolar disorder;

Translational and preclinical evidence: experimental studies in healthy participants, animal models, or cellular systems examining feeding-related entrainment, peripheral clocks, circadian misalignment, metabolic regulation, or microbial rhythmicity.

This hierarchy was used to avoid presenting findings from non-bipolar populations or preclinical models as established clinical evidence in bipolar disorder. Primary studies were used for study-level claims; reviews and meta-analyses were used to map broader mechanisms and locate additional primary reports. When a review included a primary study already cited, it was not treated as an independent replication, thereby reducing double-counting. Consensus statements informed terminology or clinical context only and were not counted as efficacy evidence.

### 2.4. Data Extraction and Narrative Synthesis

For each included study, information was extracted on authorship, publication year, country, study design, sample characteristics, diagnostic population, mood state, chrono-nutritional exposure or intervention, assessment methods, circadian and metabolic outcomes, psychiatric outcomes, main findings, and methodological limitations. Extraction was performed by one author using a prespecified form and independently verified by the second author; discrepancies were resolved by consensus against the full report.

The evidence was synthesized across the following domains: circadian and metabolic vulnerability in bipolar disorder; food intake as a synchronizer of peripheral clocks; clinically relevant forms of chrono-nutritional disruption; bidirectional relationships between mood episodes and eating timing; glucose-insulin, mitochondrial, neuroendocrine, immune-inflammatory, and microbial mechanisms; potential chrono-nutritional phenotypes; clinical assessment and digital monitoring; structured meal timing and time-restricted eating; research gaps and future directions.

No meta-analysis was performed because of substantial heterogeneity in study populations, exposure definitions, interventions, assessment methods, follow-up periods, and reported outcomes.

### 2.5. Critical Appraisal and Development of the Conceptual Model

The methodological strength and clinical relevance of the evidence were considered according to study design, sample size, diagnostic accuracy, measurement validity, control of confounding variables, temporal direction, and replication across studies. Particular attention was given to self-reported food timing, cross-sectional designs, inadequate characterization of bipolar subtype or mood phase, psychotropic medication effects, and confounding by sleep duration, physical activity, energy intake, diet quality, and body mass index.

Because this was a narrative review spanning heterogeneous designs, a single pooled risk-of-bias judgment was not applied across the entire corpus. However, direct bipolar-specific studies were appraised at study level using design-appropriate domains: selection and diagnostic characterization, exposure/outcome measurement, confounding, temporal direction, attrition where applicable, and selective reporting. 

Following thematic synthesis, the findings were integrated into a hypothesis-generating chrono-metabolic model linking circadian vulnerability, irregular food timing, central-peripheral clock misalignment, metabolic and neuroendocrine dysregulation, altered microbial rhythmicity, immune-inflammatory activation, and affective instability. Proposed pathways based mainly on indirect or preclinical evidence were explicitly distinguished from those supported by bipolar-specific clinical findings.

## 3. Bipolar Disorder as a State of Circadian and Metabolic Vulnerability

This section primarily summarizes direct bipolar-specific evidence for circadian and metabolic vulnerability; it does not establish that meal timing causes these abnormalities.

### 3.1. Sleep–Wake and Circadian Rhythm Instability

Disturbances of sleep and circadian organization are among the most consistent biological features of bipolar disorder and occur across manic, depressive, mixed, and euthymic states [4]. Mania and hypomania are typically associated with reduced sleep need, delayed sleep timing, increased nighttime activity, and marked irregularity of daily routines, whereas bipolar depression may present with insomnia, hypersomnia, delayed sleep phase, or pronounced variability in sleep duration and timing [4,6]. These abnormalities may persist during euthymia, suggesting that circadian dysregulation is not merely an epiphenomenon of acute mood episodes but may represent a trait-like component of the disorder [5,39].

Chronotype is also frequently altered in bipolar disorder. Eveningness and delayed circadian phase are more common among individuals with bipolar disorder than among healthy controls and have been associated with greater mood instability, depressive symptoms, impaired functioning, and poorer sleep quality [4,39]. A systematic review including more than 3000 individuals with bipolar disorder found evidence of biological rhythm disruption independently of current mood state and, in some studies, even among drug-naïve patients [39]. These findings support the hypothesis that altered circadian timing may contribute to illness vulnerability rather than simply result from treatment or chronicity.

The clinical course of bipolar disorder is particularly sensitive to changes in environmental and behavioral time cues. Sleep loss, shift work, transmeridian travel, seasonal changes, irregular social schedules, and disruption of daily routines may precipitate or exacerbate mood episodes in susceptible individuals [3,4]. Indeed, individual sensitivity to other environmental and meteorological fluctuations, such as meteoropathy and meteorosensitivity, represents an additional layer of constitutional vulnerability that can destabilize clinical and biological homeostasis in affective disorders. The social zeitgeber hypothesis proposes that life events capable of disrupting habitual routines can destabilize endogenous biological rhythms and thereby increase the likelihood of affective episodes [40]. The efficacy of interpersonal and social rhythm therapy, which explicitly targets the regularity of sleep, meals, activity, and interpersonal routines, provides indirect clinical support for the relevance of behavioral rhythm stabilization in bipolar disorder [40,41].

### 3.2. Central and Peripheral Clock Dysregulation

The mammalian circadian system consists of a central pacemaker in the suprachiasmatic nucleus and multiple peripheral clocks distributed across the brain and metabolic organs [8,9]. At the molecular level, circadian rhythms are generated through transcriptional-translational feedback loops involving CLOCK and BMAL1, which promote the transcription of PER and CRY genes; the encoded proteins subsequently inhibit CLOCK–BMAL1 activity and generate an approximately 24 h oscillation [9]. Additional regulatory loops involving REV-ERB and ROR nuclear receptors contribute to the stability and metabolic integration of the clock machinery [42].

Several components of the circadian system have been implicated in bipolar disorder, including altered melatonin secretion, cortisol rhythmicity, temperature regulation, clock-gene expression, and sensitivity to environmental light [5,43]. Genetic association studies have reported links between bipolar disorder and variants in circadian-related genes, although individual findings have often been inconsistent and effect sizes are generally modest [44]. Rather than reflecting dysfunction of a single clock gene, the disorder may involve impaired coordination among multiple oscillators and altered responsiveness to environmental zeitgebers [4].

This distinction is important for chrono-nutrition. Light is the dominant synchronizer of the central suprachiasmatic clock, whereas food intake strongly entrains peripheral clocks in the liver, pancreas, gastrointestinal tract, adipose tissue, and skeletal muscle [10,27]. When sleep, light exposure, activity, and food intake occur at discordant times, central and peripheral rhythms may become misaligned [24]. Individuals with bipolar disorder, whose sleep and social rhythms are already unstable, may therefore be particularly susceptible to the additional desynchronizing effects of irregular or nocturnal eating.

### 3.3. Neuroendocrine Dysregulation

The hypothalamic–pituitary–adrenal axis is closely regulated by the circadian system and displays a marked daily rhythm, with cortisol concentrations normally peaking around awakening and declining across the day [45]. Alterations in basal cortisol levels, diurnal slope, stress reactivity, and glucocorticoid feedback have been reported in bipolar disorder, although findings vary according to mood state, illness stage, medication exposure, and methodological approach [46]. Hyperactivity of the hypothalamic–pituitary–adrenal axis appears to be more pronounced during acute mood episodes, particularly mania and depression, but residual abnormalities may also persist during euthymia [46].

Meal timing can influence cortisol and metabolic rhythms, while cortisol itself affects appetite, glucose regulation, and food preference [47]. Late eating, nocturnal caloric intake, sleep restriction, and circadian misalignment may flatten or shift endocrine rhythms and increase postprandial glucose exposure [23,29]. In bipolar disorder, recurrent interaction between altered sleep timing, stress-system dysregulation, and irregular food intake may therefore amplify both neuroendocrine and metabolic instability.

Appetite-regulating hormones may also be involved. Leptin, ghrelin, insulin, and adiponectin show circadian variation and respond to meal timing, fasting duration, sleep, and energy balance [47]. Alterations in these signals have been described in bipolar disorder, particularly in association with obesity, psychotropic treatment, and metabolic syndrome [48]. It remains uncertain whether these abnormalities reflect intrinsic disease mechanisms, medication effects, lifestyle disruption, or their interaction.

### 3.4. Metabolic Comorbidity and Insulin Resistance

Metabolic abnormalities are highly prevalent in bipolar disorder. Individuals with the disorder have increased rates of overweight, obesity, metabolic syndrome, dyslipidemia, hypertension, insulin resistance, and type 2 diabetes compared with the general population [13,15]. These conditions contribute substantially to cardiovascular morbidity and premature mortality and may also be associated with a more severe psychiatric course [1].

Psychotropic medications, particularly some antipsychotics and mood stabilizers, can promote weight gain, dyslipidemia, and impaired glucose regulation [49]. Nevertheless, metabolic dysfunction in bipolar disorder cannot be attributed exclusively to pharmacotherapy. Insulin resistance and abnormalities in glucose homeostasis have also been reported in untreated or early-stage patients, suggesting that shared biological and behavioral mechanisms may contribute to the association [50]. Insulin resistance has been associated with chronicity, rapid cycling, treatment resistance, cognitive impairment, and poorer functional outcomes, although the direction of these relationships remains incompletely defined [50].

The relationship between psychiatric and metabolic disorders is likely bidirectional. Depression, chronic stress, sleep disruption, physical inactivity, inflammatory activation, and unhealthy dietary patterns may increase the risk of type 2 diabetes, whereas insulin resistance and diabetes may adversely affect brain function, cognition, and mood regulation. This bidirectionality is particularly relevant to chrono-nutrition because eating at biologically adverse times can worsen glucose tolerance even when caloric intake is unchanged [23]. Therefore, meal timing may represent a behavioral pathway through which circadian instability is translated into metabolic burden.

### 3.5. Mitochondrial Dysfunction and Metabolic Inflexibility

Mitochondrial dysfunction is increasingly recognized as a relevant component of bipolar disorder pathophysiology [2,43]. Reported abnormalities include impaired oxidative phosphorylation, altered adenosine triphosphate production, increased oxidative stress, disturbances in calcium signaling, and changes in mitochondrial morphology and gene expression [43,51]. These alterations may affect neuronal excitability, synaptic plasticity, cellular resilience, and the capacity to adapt energy production to changing physiological demands [2].

A state-dependent model of energy dysregulation has been proposed in which mania is associated with increased energy mobilization and hypermetabolic activity, whereas bipolar depression is characterized by reduced energy availability, impaired substrate utilization, and diminished metabolic flexibility [34,51]. Although this model remains partly hypothetical, it provides a biologically plausible framework for integrating fluctuations in sleep, activity, appetite, and mood.

Metabolic flexibility refers to the capacity to switch efficiently between carbohydrate and lipid oxidation according to feeding, fasting, and energy demand [52]. Irregular eating schedules, prolonged nighttime intake, short fasting intervals, and recurrent circadian misalignment can impair this transition and reduce temporal coordination between nutrient availability and cellular energy production [10,24]. In a disorder already characterized by possible mitochondrial and bioenergetic vulnerability, chrono-nutritional disruption may further reduce the ability to adapt to shifts between feeding and fasting states.

### 3.6. Inflammation, Oxidative Stress, and Lipid Regulation

Low-grade immune-inflammatory activation has been repeatedly reported in bipolar disorder, including altered concentrations of C-reactive protein, interleukin-6, tumor necrosis factor-α, and other inflammatory mediators [53,54]. Although inflammatory findings vary across studies, mood phases, and treatment conditions, the overall evidence supports an interaction among immune activation, oxidative stress, mitochondrial dysfunction, and neuronal signaling [43,54].

Circadian rhythms regulate immune-cell trafficking, cytokine production, redox balance, and lipid metabolism [55]. Conversely, inflammation can interfere with clock-gene expression and weaken circadian amplitude [56]. Meal timing may modify this relationship because late-night eating, high postprandial glucose exposure, and recurrent feeding during the biological night can promote oxidative and inflammatory responses [47]. Disrupted temporal organization of food intake may therefore contribute to a self-reinforcing interaction among circadian misalignment, metabolic dysfunction, and inflammation.

Lipid metabolism is also under circadian control. Daily rhythms influence hepatic lipid synthesis, fatty-acid oxidation, lipoprotein metabolism, and lipid-droplet dynamics [57]. Circadian and lipid abnormalities may converge in bipolar disorder, where dyslipidemia is common and several lipid-related pathways have been implicated in neuronal membrane function, signaling, and energy regulation [57]. However, direct evidence linking meal timing, lipid rhythmicity, and mood instability in bipolar disorder remains scarce.

### 3.7. State- and Trait-Dependent Interactions

The biological abnormalities associated with bipolar disorder are unlikely to be static. Sleep, cortisol, inflammatory activity, mitochondrial function, appetite, and energy expenditure may vary across mania, depression, mixed states, and euthymia [4,51]. Some alterations may represent enduring vulnerability markers, whereas others may emerge or intensify during acute episodes.

This distinction has direct implications for chrono-nutrition. During mania or hypomania, reduced sleep need, hyperactivity, impulsivity, and diminished attention to routine may lead to skipped meals, irregular intake, or nocturnal eating. During bipolar depression, delayed sleep timing, fatigue, emotional eating, reduced motivation, and hypersomnia may favor breakfast omission, late first caloric intake, or concentration of energy consumption in the evening. Consequently, the same chrono-nutritional intervention may not be equally appropriate across all mood states.

The available evidence therefore supports viewing bipolar disorder as a condition in which circadian, metabolic, neuroendocrine, mitochondrial, and inflammatory vulnerabilities converge. These systems are strongly influenced by the timing and regularity of behavioral inputs, including food intake. Chrono-nutritional disruption may consequently represent not an isolated lifestyle factor, but a potential amplifier of an already unstable biological network.

## 4. Food Intake as a Circadian Zeitgeber

Findings in this section derive mainly from controlled human studies outside bipolar disorder and from preclinical models. Their application to bipolar disorder is inferential.

### 4.1. Central and Peripheral Circadian Organization

The circadian system coordinates physiology and behavior with the approximately 24 h environmental cycle through a hierarchical network of central and peripheral oscillators [8,9]. The suprachiasmatic nucleus of the hypothalamus functions as the principal central pacemaker and is entrained predominantly by light signals transmitted from the retina [8]. It subsequently coordinates peripheral clocks through neural, endocrine, autonomic, behavioral, and temperature-related signals [8,10].

Peripheral oscillators are present in metabolically active tissues, including the liver, pancreas, gastrointestinal tract, adipose tissue, and skeletal muscle [10,58]. These clocks regulate tissue-specific rhythms in glucose handling, insulin secretion, lipid metabolism, nutrient absorption, gastrointestinal motility, energy expenditure, and immune activity [10,59]. Although peripheral clocks are normally coordinated with the central pacemaker, they are also strongly responsive to the timing of food intake [27,60].

Food should therefore be considered not only a source of energy and nutrients but also a recurring temporal signal. Whereas light primarily entrains the central clock, feeding–fasting cycles provide timing information to peripheral metabolic tissues [27,61]. Experimental displacement of feeding time can shift peripheral oscillators while leaving the phase of the suprachiasmatic nucleus relatively unchanged, thereby producing internal circadian misalignment [27]. However, sensitivity to food timing is tissue-specific, and the liver and adipose tissue appear more readily entrainable than some other organs [62,63].

### 4.2. Feeding-Fasting Cycles and Peripheral Clock Entrainment

The alternation between feeding and fasting generates rhythmic changes in circulating glucose, insulin, amino acids, free fatty acids, bile acids, gut-derived hormones, and cellular energy sensors [10,60]. These signals interact with the molecular clock through pathways involving insulin signaling, cyclic adenosine monophosphate, glucocorticoids, adenosine monophosphate-activated protein kinase, mechanistic target of rapamycin, and nicotinamide adenine dinucleotide-dependent sirtuins [10,64].

During the feeding phase, insulin and nutrient-sensitive pathways promote glucose utilization, glycogen synthesis, lipogenesis, and protein synthesis, whereas fasting favors lipolysis, fatty-acid oxidation, ketogenesis, autophagy, and cellular maintenance [65]. Regular alternation between these states contributes to temporal separation of anabolic and catabolic processes [65]. Repeated eating across an extended daily interval may shorten the fasting period and reduce this metabolic separation, particularly when caloric intake continues into the biological night [10,63].

Restricted-feeding experiments initially demonstrated that changing food availability could uncouple hepatic and other peripheral oscillators from the central pacemaker [27]. Subsequent evidence has shown that meal timing can also alter selected human circadian rhythms. In a controlled laboratory study, delaying meals by five hours delayed the rhythm of glucose and adipose-tissue PER2 expression without significantly shifting melatonin or cortisol rhythms, suggesting preferential effects on peripheral rather than central circadian markers [66]. Human glucose rhythms and subjective hunger can also anticipate a recurrent meal schedule, supporting the presence of food-related temporal adaptation [67].

These findings are particularly relevant when sleep–wake timing and food timing become dissociated. Eating according to external schedules rather than biological time may expose peripheral organs to nutrients during phases in which their metabolic capacity is reduced [24]. In individuals with bipolar disorder, irregular sleep and activity patterns may increase the likelihood that meals occur at inconsistent circadian phases, even when their clock time appears superficially similar from one day to another.

**Figure 2:**
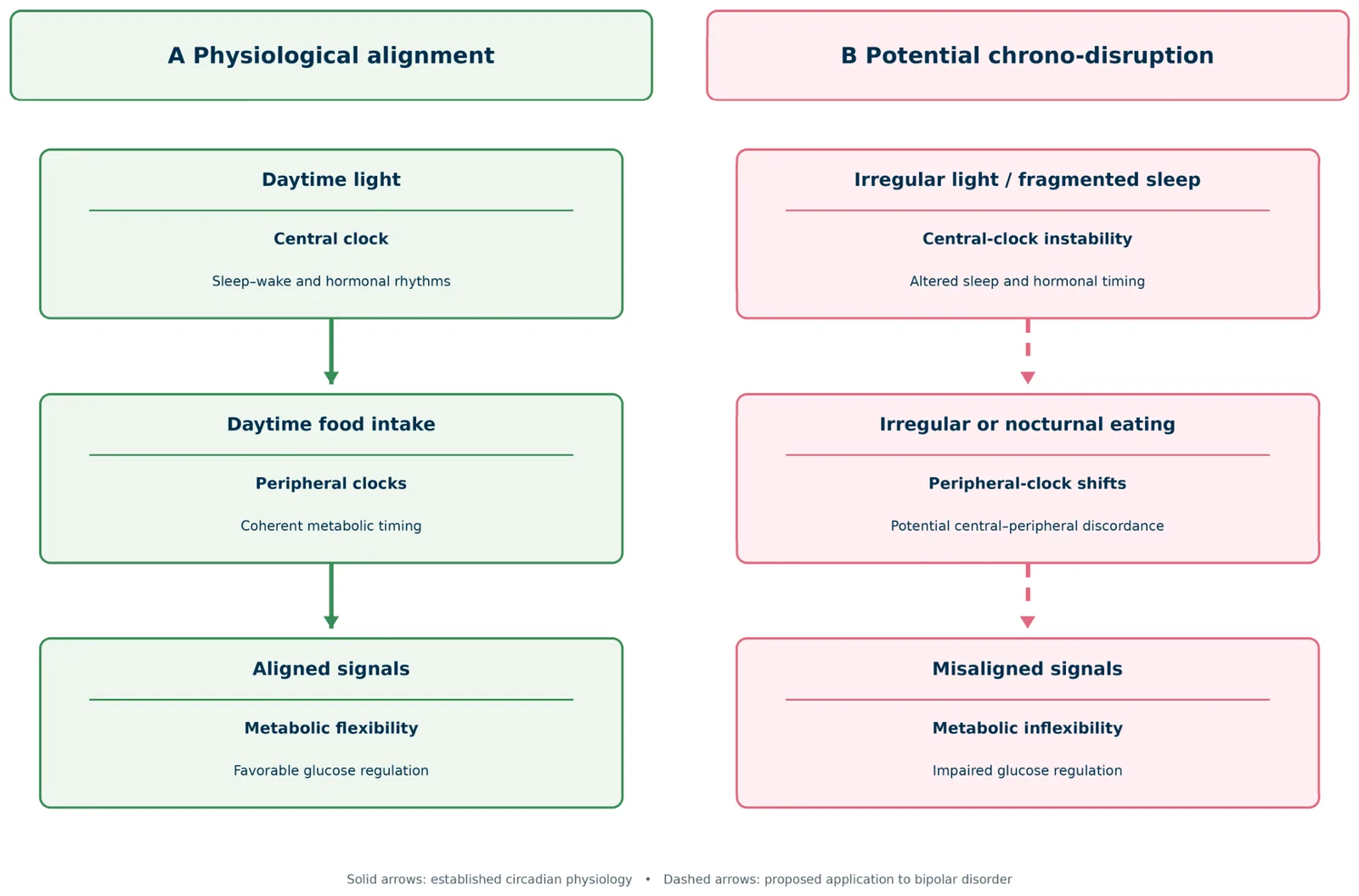
Circadian alignment vs. chrono-disruption of central and peripheral pacemakers. Note. Panel (**A**) (Physiological Alignment/Synchronization): Under healthy conditions, daytime photic signals entrain the suprachiasmatic nucleus (SCN), which regulates sleep–wake cycles and central hormonal rhythms (e.g., cortisol, melatonin). Food intake restricted to the active diurnal phase acts as a coherent zeitgeber, synchronizing peripheral clocks (liver, pancreas, skeletal muscle, adipose tissue) and promoting optimal glucose tolerance and metabolic flexibility. Panel (**B**) (Chrono-Disruption in Bipolar Disorder): Exposure to nocturnal artificial light, fragmented sleep, and erratic, nocturnal eating patterns (night eating) decouple peripheral clocks from the central SCN pacemaker. Nutrients consumed during the subjective biological rest phase (overlapping with elevated melatonin levels) trigger internal desynchronization, leading to postprandial glucose intolerance, blunted insulin secretion, and ectopic lipid accumulation.

The systemic contrast between physiological circadian alignment (synchrony) and the chrono-disruption characteristic of bipolar disorder (desynchrony) is schematically illustrated in Figure 2.

### 4.3. Circadian Variation in Postprandial Metabolism

Human metabolic responses to food vary across the biological day [24,59]. Glucose tolerance, pancreatic β-cell responsiveness, diet-induced thermogenesis, and substrate oxidation are generally more favorable during the earlier active phase than during the evening or biological night [23,59]. Consequently, an identical meal may produce different postprandial glucose and insulin responses depending on when it is consumed [24].

Circadian misalignment can impair glucose tolerance independently of sleep duration and caloric intake [23,29]. Controlled studies have shown that delayed eating schedules may increase mean glucose concentrations, reduce carbohydrate or fat oxidation, alter cortisol profiles, and decrease thermic responses to food [68,69,70]. These effects indicate that metabolic consequences arise not solely from dietary quantity or composition but also from temporal discordance between nutrient intake and endogenous physiology.

The relationship between melatonin and glucose metabolism is especially relevant to late eating. Endogenous melatonin concentrations rise during the evening and biological night, when insulin secretion and glucose tolerance are reduced [71]. Randomized crossover studies have shown that consuming dinner close to the melatonin rise can impair glucose tolerance, particularly among carriers of the common MTNR1B glucose-risk allele [71,72]. Thus, the metabolic impact of meal timing may differ according to individual circadian phase and genetic susceptibility rather than clock time alone.

This distinction is clinically important because late eating cannot be defined adequately by a universal hour. A meal consumed at 21:00 may occur at very different biological phases in an early chronotype and an evening chronotype [22]. Chrono-nutritional assessment should therefore consider the interval between food intake and sleep onset, dim-light melatonin onset, habitual activity timing, and the midpoint of sleep, whenever feasible [22,73].

### 4.4. Meal Timing, Regularity, and Daily Energy Distribution

Chrono-nutrition encompasses several related but distinct dimensions: meal timing, meal frequency, regularity, duration of the daily eating window, fasting interval, breakfast consumption, nocturnal eating, and distribution of caloric intake across the day [22,26]. These dimensions should not be collapsed into a single exposure because they may influence metabolism through partially different pathways.

Meal regularity provides a predictable temporal signal to peripheral tissues, whereas day-to-day variability may weaken the alignment between anticipated nutrient availability and metabolic responses [74]. Irregular meal patterns have been associated with less favorable cardiometabolic profiles, although much of the available evidence is observational and susceptible to confounding by sleep, occupation, diet quality, physical activity, and socioeconomic factors [22,74].

The timing of energy distribution may also be relevant. Consuming a larger proportion of total daily energy earlier in the day has frequently been associated with better weight and glycemic outcomes than concentrating intake in the evening, although findings are not entirely consistent across populations and intervention designs [22,75]. Late habitual caloric intake has been associated with poorer glucose tolerance even after adjustment for body weight, energy intake, and diet composition in individuals with impaired glucose regulation [76].

Breakfast omission may represent either a distinct behavioral exposure or a marker of delayed circadian timing. Its metabolic implications depend on whether breakfast is skipped as part of an intentional, stable eating window or within an irregular pattern accompanied by late-night intake and reduced sleep [22]. Therefore, the clinical meaning of breakfast skipping cannot be determined without considering the overall eating window, chronotype, energy distribution, and timing of the last meal.

### 4.5. Late and Nocturnal Eating

Late eating refers broadly to caloric intake occurring toward the end of the waking period, whereas nocturnal eating implies intake during the habitual sleep period or biological night [22]. Both behaviors can increase overlap between nutrient intake, elevated melatonin concentrations, reduced glucose tolerance, and the circadian phase normally allocated to fasting and cellular restoration [24,75].

Controlled studies indicate that later meals may increase postprandial glucose exposure and reduce fat oxidation even when caloric intake, dietary composition, sleep timing, and duration of the eating window are held constant [69,77]. Late eating has also been associated experimentally with increased hunger, reduced waking energy expenditure, and adipose-tissue molecular changes favoring lipid storage [30].

Nevertheless, these results should not be generalized uncritically to bipolar disorder. Late or nocturnal eating may arise from evening chronotype, insomnia, shift work, medication-induced appetite, emotional eating, reduced daytime structure, or acute mood symptoms. Each pathway may carry different psychiatric and metabolic implications. In addition, most experimental meal-timing studies have involved metabolically healthy adults or individuals with overweight, obesity, prediabetes, or type 2 diabetes rather than patients with bipolar disorder [22].

### 4.6. Social Jet Lag and Eating Jet Lag

Social jet lag describes the discrepancy between biological and socially imposed timing, commonly estimated from differences in sleep timing between workdays and free days [78]. A related construct, eating jet lag, refers to day-to-day differences in meal timing, particularly between weekdays and weekends [79]. These discrepancies may produce repeated shifts in the timing of metabolic signals without sufficient time for stable adaptation [79].

Later chronotypes tend to eat later, skip breakfast more frequently, and show greater variability in meal timing than earlier chronotypes [80]. Chronotype alone does not determine chrono-nutritional risk. The degree of alignment between preferred timing, actual behavior, light exposure, occupational demands, and sleep schedule may be more important than absolute clock time [22].

This framework may be especially pertinent to bipolar disorder, in which social rhythm irregularity and eveningness are common [5,46]. Recurrent discrepancies among sleep timing, social obligations, and food intake could increase instability in peripheral metabolic rhythms. Direct evidence demonstrating that eating jet lag predicts mood episodes in bipolar disorder is currently lacking, however, and this relationship should be considered a priority for prospective research.

### 4.7. Time-Restricted Eating as a Circadian Intervention

Time-restricted eating limits daily caloric intake to a consistent interval without necessarily prescribing caloric restriction or specific macronutrient composition [81]. Its proposed mechanisms include reinforcement of feeding–fasting rhythms, reduction in late-night intake, increased fasting duration, improved alignment between nutrient intake and circadian metabolic capacity, and indirect changes in energy consumption [81,82].

Systematic reviews and meta-analyses suggest that time-restricted eating may produce modest improvements in body weight, fasting glucose, insulin sensitivity, and other cardiometabolic outcomes, although findings vary substantially according to intervention duration, eating-window timing, adherence, comparator conditions, and concurrent caloric restriction [82,83]. Earlier eating windows generally appear more favorable for glycemic control than later windows, supporting the relevance of circadian alignment rather than fasting duration alone [84].

The apparent benefits of time-restricted eating should nevertheless be interpreted cautiously. Some effects may result from unintended reductions in energy intake, improved dietary regularity, or reduced evening snacking rather than direct clock resetting [81,82]. Furthermore, optimal eating-window duration and timing remain uncertain, and evidence concerning long-term adherence, psychiatric safety, medication interactions, and applicability across different chronotypes is limited.

In bipolar disorder, structured and consistent meal timing may be conceptually more appropriate than rigid fasting prescriptions. Restrictive protocols could be unsuitable during mania, mixed states, active eating disorders, pregnancy, diabetes treated with glucose-lowering medication, or when psychotropic drugs must be taken with food. Accordingly, time-restricted eating should currently be regarded as an investigational adjunct rather than an established treatment for bipolar disorder.

### 4.8. Relevance to Bipolar Disorder

The temporal organization of food intake may intersect with bipolar disorder at several levels. Irregular meals can weaken peripheral temporal cues; eating during the biological night can worsen glucose regulation; shortened fasting intervals may impair metabolic switching; and inconsistent schedules may intensify misalignment among sleep, activity, hormonal rhythms, and nutrient availability [10,24].

These effects may be clinically relevant in a population already characterized by circadian instability, metabolic comorbidity, medication-related appetite changes, and vulnerability to disrupted daily routines [1,4]. The direct evidence remains insufficient to establish that chrono-nutritional disruption causes mood episodes or that correcting meal timing independently improves bipolar outcomes.

The most defensible current interpretation is therefore that food timing represents a potentially modifiable behavioral input within a broader circadian and metabolic system. Its effect is likely to depend on mood phase, chronotype, sleep timing, medication, diet quality, metabolic status, and the regularity of other daily routines. This multidimensional perspective provides the basis for examining the specific forms of chrono-nutritional disruption most relevant to bipolar disorder.

## 5. Forms of Chrono-Nutritional Disruption Relevant to Bipolar Disorder

Direct bipolar-specific findings are available chiefly for night eating and related chronotype/sleep correlates. Evidence for other categories is indirect or absent, as indicated in Table 1.

### 5.1. Irregular Meal Timing

Irregular meal timing is a clinically plausible but insufficiently investigated form of chrono-nutritional disruption in bipolar disorder. It includes substantial day-to-day variability in the timing of meals, omission or postponement of planned meals, unpredictable eating episodes, and poor coordination between food intake, sleep, activity, and medication schedules [17,22]. Unlike consistently early or late eating, irregularity provides peripheral clocks with unstable temporal signals and may repeatedly alter the phase relationship between nutrient availability and endogenous metabolic rhythms [74,79].

This pattern may be especially relevant to bipolar disorder because instability of daily routines is a recognized feature of the illness [4,85]. Individuals with bipolar disorder may experience changes in sleep, activity, social engagement, appetite, and reward sensitivity both during acute episodes and between episodes [4,86]. As meals are embedded within these daily routines, disruption of social rhythms may also reduce the regularity of eating behavior [40,41].

The available evidence suggests that people with bipolar disorder may exhibit both greater circadian disruption and more disturbed eating behavior than healthy controls, although direct associations between the two dimensions have not been consistently demonstrated [87]. Accordingly, irregular meal timing should currently be regarded as a plausible component of broader behavioral rhythm instability rather than as an independently established predictor of bipolar relapse.

### 5.2. Delayed First Caloric Intake and Breakfast Skipping

Breakfast skipping is common among individuals with evening chronotype and may reflect delayed circadian timing, shortened morning wakefulness, reduced appetite after awakening, or continuation of food intake late into the previous evening [22,80]. Its clinical interpretation depends on the broader temporal pattern. A consistently delayed first meal within an otherwise regular eating window differs substantially from erratic breakfast omission followed by prolonged daytime fasting and compensatory evening intake [22].

In bipolar depression, hypersomnia, delayed sleep timing, fatigue, psychomotor slowing, and reduced motivation may postpone the first daily meal [4,6]. During mania or hypomania, breakfast may instead be missed because of reduced attention to bodily needs, increased goal-directed activity, distractibility, or a disrupted sleep–wake schedule [4,88]. These possible pathways remain clinically plausible but have not been adequately evaluated in prospective studies of bipolar disorder.

Breakfast omission is therefore unlikely to represent a uniform risk behavior. Its relevance should be assessed in relation to wake time, chronotype, total energy intake, medication requirements, fasting duration, and the timing of the final caloric intake on the preceding day [22]. In clinical practice, the regularity and circadian position of the first meal may be more informative than the conventional label of breakfast alone.

### 5.3. Late Eating and Evening Caloric Concentration

Late eating refers to the consumption of a substantial proportion of daily energy near the end of the waking period or close to habitual sleep onset [22]. Evening chronotype is associated with later food intake, a delayed temporal distribution of energy, more frequent breakfast omission, and greater variability between workdays and free days [80,89]. Because eveningness is common in bipolar disorder, delayed eating may represent an important point of convergence between circadian preference and metabolic vulnerability [39,90].

The metabolic relevance of late eating is supported by experimental evidence showing that nutrient intake near the biological night can impair glucose tolerance, alter substrate oxidation, increase hunger, and favor energy storage [30,75]. Evidence that habitual late eating independently worsens mood course in bipolar disorder remains insufficient.

A delayed eating pattern may also be adaptive when aligned with a stable late chronotype, whereas forced eating at an earlier social time may not always improve internal alignment [22]. The clinically important issue may therefore be the degree of concordance among food timing, sleep phase, melatonin onset, activity, and social obligations rather than late clock time alone.

### 5.4. Night Eating and Nocturnal Ingestion

Night eating syndrome is characterized by recurrent evening hyperphagia, nocturnal awakenings accompanied by food intake, or both, with awareness and recall of the episodes [91,92]. It should be distinguished from occasional late snacking, binge-eating disorder, and sleep-related eating disorder [91,92].

Night eating appears to be more frequent among individuals with bipolar disorder than among healthy controls [93]. In one bipolar sample, night eating was associated with greater anxiety, more severe insomnia, poorer sleep quality, lower functioning, and a stronger evening preference [93]. More recent evidence in euthymic bipolar I disorder has also linked night-eating symptoms with chronotype, seasonality, and sleep quality, with sleep disturbance potentially mediating part of this relationship [94].

These associations suggest that night eating may identify a clinically meaningful subgroup characterized by overlapping circadian, sleep, affective, and metabolic disturbances. Nevertheless, the available studies are predominantly cross-sectional and cannot establish whether night eating contributes to mood instability, results from it, or reflects shared underlying vulnerability [93,94].

Night eating may also occur in patients with obesity or candidates for bariatric surgery, in whom bipolar-spectrum conditions and other psychiatric comorbidities may be overrepresented [95]. This overlap reinforces the need to distinguish bipolar-specific effects from those related to obesity, sleep disruption, emotional distress, or comorbid eating disorders.

### 5.5. Prolonged, Inconsistent, or Unplanned Fasting

Fasting is not inherently equivalent to chrono-nutritional disruption. A stable, clinically supervised eating window differs from unplanned or highly variable periods without food resulting from mood symptoms, chaotic routines, financial constraints, substance use, or reduced self-care [22,65].

During mania or hypomania, individuals may remain awake and active for prolonged periods while eating inconsistently or inadequately. In bipolar depression, reduced appetite, psychomotor slowing, hypersomnia, and diminished motivation may also lengthen fasting intervals [88]. Conversely, atypical depressive presentations may involve increased appetite and hyperphagia rather than fasting [88].

The metabolic consequences of fasting depend on duration, timing, regularity, baseline nutritional status, medication use, and the pattern of subsequent refeeding [65]. In bipolar disorder, abrupt or prolonged fasting may be clinically problematic when associated with sleep deprivation, dehydration, substance use, eating-disorder psychopathology, diabetes, or medications that should be taken with food. It should therefore not be assumed that a longer fasting interval is uniformly beneficial.

### 5.6. Extended Eating Windows and Recurrent Snacking

An extended eating window occurs when caloric intake is distributed across most of the waking day and may continue into the habitual sleep period [96]. Frequent snacking and prolonged daily access to food can reduce the duration of the fasting phase and blur the temporal separation between anabolic and catabolic processes [10,81].

In bipolar disorder, extended eating windows may arise through several mechanisms, including insomnia, prolonged nighttime wakefulness, medication-related hunger, boredom, emotional regulation through food, and reduced structure of daily activities [17,97]. Sedating psychotropic medications taken in the evening may also coincide with increased appetite or reduced inhibitory control, although direct evidence concerning their effects on the timing rather than quantity of intake remains limited.

This distinction is important because medication-associated weight gain has generally been studied in terms of appetite, caloric intake, body weight, and metabolic parameters rather than eating-window duration [49]. Future studies should therefore determine whether psychotropic treatment modifies not only how much patients eat but also when eating occurs.

### 5.7. Uneven Daily Caloric Distribution

Chrono-nutritional disruption may involve a disproportionate concentration of energy intake in the evening or night, even when total daily caloric intake is not excessive [22,75]. This pattern can result from delayed first intake, skipped daytime meals, evening hyperphagia, or nocturnal eating.

In bipolar disorder, an uneven caloric distribution may fluctuate with mood polarity. Manic or hypomanic states may be associated with reduced or distracted eating during daytime activity followed by opportunistic intake later in the day. Bipolar depression may promote either reduced overall intake or delayed compensatory eating after prolonged inactivity or hypersomnia. These patterns are clinically plausible but remain poorly characterized.

Because identical total caloric intake may have different metabolic effects according to its temporal distribution, future bipolar studies should report the proportion of energy consumed during predefined circadian intervals rather than relying only on total intake or conventional meal labels [22,24].

### 5.8. Eating Jet Lag and Weekday-Weekend Variability

Eating jet lag refers to differences in meal timing between workdays and free days and is conceptually related to social jet lag [79]. Repeated shifts in breakfast, lunch, dinner, and eating-window timing may expose peripheral clocks to alternating schedules and impair stable entrainment [79,98].

Individuals with evening chronotype and those whose preferred rhythms conflict with occupational or family schedules may be particularly susceptible [80]. Bipolar disorder may amplify this mismatch because patients often show delayed chronotype, irregular sleep, unemployment or changing work participation, and fluctuations in social activity across mood states [4,39].

Low social-rhythm regularity has been prospectively associated with the first onset of bipolar-spectrum episodes in vulnerable individuals [7]. Whether variability in meal timing contributes independently to this risk has not been established. Eating jet lag should therefore be considered a candidate behavioral marker requiring direct longitudinal evaluation.

### 5.9. Shift Work, Travel, and Environmentally Imposed Misalignment

Shift work, night work, transmeridian travel, and irregular occupational schedules can desynchronize sleep, light exposure, activity, and food intake [99]. Meals consumed during night shifts occur at a circadian phase characterized by reduced glucose tolerance and altered metabolic responsiveness [24].

Sleep loss, travel across time zones, and disruption of habitual schedules may precipitate mood symptoms in individuals with bipolar disorder [3,4]. Food timing may contribute to this vulnerability because eating frequently remains aligned with work or destination time rather than with the individual’s internal circadian phase.

The specific contribution of meal timing during shift work or travel has not been isolated in bipolar disorder. Nevertheless, these contexts provide clinically relevant natural models of combined circadian and chrono-nutritional misalignment and warrant targeted preventive strategies.

### 5.10. Mood-State-Specific Chrono-Nutritional Patterns

Chrono-nutritional disruption is unlikely to be uniform across bipolar mood states. Mania and hypomania may involve reduced sleep need, elevated activity, impulsivity, distractibility, heightened reward sensitivity, and decreased adherence to routine, potentially resulting in skipped meals, irregular eating, nocturnal intake, or prolonged eating windows [86,88]. Bipolar depression may instead involve delayed sleep, hypersomnia, low motivation, emotional eating, appetite loss, increased appetite, or concentration of food intake later in the day [6,100].

Mixed features may be particularly destabilizing because depressive distress can coexist with agitation, insomnia, impulsivity, and increased activation [88]. This combination may promote irregular, emotionally driven, or nocturnal eating, although direct evidence remains sparse. Rapid cycling may similarly be accompanied by recurrent changes in appetite, sleep, and eating schedules as mood polarity shifts.

Accordingly, chrono-nutritional assessment should record current mood state, recent polarity changes, sleep timing, appetite direction, psychomotor activity, and medication modifications. A pattern observed during acute mania should not automatically be interpreted as a stable trait, and an intervention appropriate during euthymia may be unsafe or impractical during an acute episode.

### 5.11. Reward Sensitivity, Impulsivity, and Comorbid Eating Disorders

Reward-system hypersensitivity and impulsivity are implicated in bipolar-spectrum disorders and may interact with circadian disruption [86]. Food is a potent reward stimulus, and reduced inhibitory control, emotional dysregulation, or heightened reward seeking may influence both the amount and timing of intake [97].

Eating disorders are clinically relevant comorbidities in bipolar disorder. Meta-analytic evidence indicates meaningful overlap with binge-eating disorder and bulimia nervosa, although estimates vary substantially across studies [101]. This comorbidity may complicate chrono-nutritional interpretation because late eating, fasting, binge episodes, and irregular meals can reflect an eating disorder rather than circadian misalignment alone.

Screening for binge eating, compensatory restriction, night eating, and other disordered eating behaviors is therefore essential before recommending time-restricted or fasting-based interventions. In patients with active eating-disorder psychopathology, increasing dietary restriction may worsen symptoms and should not be considered a routine chrono-nutritional strategy.

### 5.12. An Integrated Clinical Interpretation

The principal forms of chrono-nutritional disruption relevant to bipolar disorder include irregular meal timing, delayed first intake, late or nocturnal eating, unplanned fasting, extended eating windows, uneven caloric distribution, and weekday-weekend variability. These patterns frequently overlap and may reflect a common breakdown in the temporal organization of daily behavior.

Their clinical significance cannot be inferred from clock time alone. The same behavior may have different implications depending on chronotype, sleep phase, mood state, medication, metabolic status, total dietary intake, and comorbid eating psychopathology. Chrono-nutritional disruption should therefore be conceptualized as a multidimensional and state-sensitive phenotype rather than as a single exposure.

Current evidence is strongest for the increased occurrence and clinical correlates of night eating in bipolar disorder [93,94]. Evidence concerning meal irregularity, eating jet lag, caloric distribution, and fasting remains largely indirect or hypothesis-generating. This imbalance highlights the need for objective, longitudinal studies capable of determining whether specific eating-timing patterns precede mood destabilization or merely accompany it.

The principal forms of chrono-nutritional disruption potentially relevant to bipolar disorder, together with their possible determinants, biological implications, and current level of evidence, are summarized in Table 1.

## 6. Bidirectional Links Between Mood Episodes and Eating Timing

Evidence status: Bipolar symptoms plausibly alter eating timing, but evidence that altered eating timing subsequently drives episodes is largely cross-sectional or mechanistic; the proposed bidirectionality remains a research hypothesis.

### 6.1. Mood Episodes as Drivers of Eating-Timing Disruption

The relationship between bipolar disorder and eating behavior is intrinsically bidirectional. Mood episodes can alter appetite, food reward, self-care, sleep timing, daily activity, and executive control, thereby changing not only how much and what individuals eat but also when eating occurs [97,102]. In turn, disrupted eating schedules may affect glucose regulation, appetite signaling, sleep, and circadian organization, potentially reinforcing the biological conditions associated with affective instability.

Eating behavior in bipolar disorder is not characterized by a single stable pattern. Instead, appetite and food intake may fluctuate across mood states, with hypomanic and manic episodes more frequently associated with reduced appetite, hypophagia, irregular intake, and weight loss, whereas depressive episodes, particularly those with atypical features, may involve hyperphagia, carbohydrate craving, binge eating, and weight gain [97,103]. Melancholic depressive presentations may instead involve reduced appetite and weight loss, highlighting substantial heterogeneity even within the same polarity [104].

These state-dependent changes may disrupt the temporal organization of eating through different pathways. During mania, reduced sleep need, psychomotor activation, distractibility, and intense goal pursuit may displace regular meals and extend wakefulness into the biological night. During depression, delayed awakening, hypersomnia, anhedonia, reduced motivation, and emotional eating may postpone the first daily intake and concentrate energy consumption later in the day. Thus, different mood states may produce similar chrono-nutritional disruption through distinct behavioral mechanisms [19].

### 6.2. Mania, Goal Pursuit, and Neglect of Homeostatic Cues

Mania is associated with heightened behavioral activation, increased energy, intensified goal pursuit, and exaggerated responses to reward-related cues [35]. These features may reduce attention to internal homeostatic signals such as hunger, fatigue, and satiety, particularly when food intake competes with highly salient activities.

The behavioral activation system model proposes that individuals with bipolar disorder show increased sensitivity to incentives and excessive activation following perceived goal progress [35]. Longitudinal research has further suggested that reward sensitivity may contribute to mood episode recurrence in bipolar disorder [105]. Although these studies did not specifically assess meal timing, their findings provide a plausible behavioral framework through which manic activation could lead to delayed, omitted, or poorly structured meals.

The effect of reward sensitivity on eating is unlikely to be uniform. Heightened reward responsivity could increase attraction to highly palatable foods, impulsive snacking, or substance-associated caloric intake in some individuals, while intense engagement in non-food goals could suppress or postpone eating in others. Direct ecological studies are needed to determine whether reward activation predicts specific changes in eating timing during emerging mania.

### 6.3. Bipolar Depression, Appetite Phenotypes, and Temporal Delay

Bipolar depression includes clinically distinct appetite and weight phenotypes. Some patients experience appetite loss and weight reduction, whereas others develop increased appetite, food craving, hypersomnia, and weight gain [97,103]. These patterns may correspond to different biological and chrono-nutritional profiles.

A low-appetite depressive phenotype may involve reduced meal frequency, prolonged fasting, and diminished responsiveness to food cues. By contrast, a high-appetite phenotype may involve frequent eating, evening hyperphagia, comfort eating, or binge-type consumption [97]. Characterizing eating behavioral phenotypes may therefore be more informative than treating appetite change as a single depressive symptom.

Sex may further modify these associations. Women with bipolar disorder have been reported to experience higher rates of appetite and weight changes during depressive episodes and greater comorbidity with eating disorders than men [102,106]. These differences may reflect interactions among mood polarity, reproductive hormones, body-image concerns, medication effects, and social determinants of eating behavior, although specific evidence concerning meal timing remains limited.

### 6.4. Mixed Features and Competing Motivational States

Mixed episodes and depressive episodes with mixed features combine depressive affect with activation, agitation, racing thoughts, irritability, or reduced sleep. This coexistence may generate particularly unstable eating patterns because low mood or emotional distress can occur alongside increased impulsivity and prolonged wakefulness.

In such states, patients may alternate between reduced daytime intake and impulsive evening or nocturnal eating. Food may also be used transiently to modulate agitation, anxiety, or dysphoria. However, the chrono-nutritional characteristics of mixed states have not been directly investigated, and current interpretations remain hypothesis-generating.

This represents an important research gap because mixed features are clinically associated with greater symptom burden, suicidality, substance use, and treatment complexity. Objective assessment of eating timing during mixed states could clarify whether meal irregularity serves as an early behavioral marker of destabilization.

### 6.5. Appetite-Regulating Hormones and Mood-State Dependence

The bidirectional relationship between mood and eating may also involve altered peripheral appetite signals. A systematic review and meta-analysis found that leptin and insulin concentrations were elevated in euthymic individuals with bipolar disorder, whereas findings for other appetite-related hormones varied according to mood state, body composition, and medication exposure [107].

Ghrelin findings are similarly heterogeneous. In one study, both acylated and total ghrelin levels were reduced in medicated patients recovering from a manic episode, although subjective hunger did not differ significantly from controls [108]. These results suggest that biological appetite signals and conscious hunger may not always move in parallel in bipolar disorder.

Because leptin, insulin, and ghrelin are influenced by sleep, fasting duration, meal timing, adiposity, and circadian phase, altered concentrations cannot be attributed solely to the psychiatric disorder [107]. Future studies should measure these hormones together with objectively recorded food timing and mood state rather than assessing them as isolated biomarkers.

### 6.6. Sleep as a Mediator Between Mood and Eating Timing

Sleep disturbance may mediate part of the relationship between mood symptoms and eating behavior. Delayed sleep onset, insomnia, nocturnal awakenings, and prolonged wakefulness increase opportunities for late eating, whereas hypersomnia and delayed awakening can postpone the first daily meal. Conversely, evening caloric intake may alter glucose regulation, body temperature, gastrointestinal activity, and sleep quality.

The direction of these associations may vary across mood states. During mania, reduced sleep need may expand the daily eating window or produce unstructured nocturnal intake. During bipolar depression, hypersomnia may compress daytime intake while shifting eating toward the evening. In both cases, sleep disruption may weaken the temporal distinction between feeding and fasting.

Studies of night eating in bipolar disorder support this overlap, as night-eating symptoms have been associated with insomnia, poorer sleep quality, anxiety, eveningness, and reduced functioning [93,94]. Nevertheless, longitudinal data are required to determine whether sleep disruption precedes changes in eating timing or whether both emerge simultaneously during mood destabilization.

### 6.7. Emotional Regulation, Food Reward, and Binge-Type Eating

Food may serve as a short-term regulator of negative affect, particularly when distress tolerance and inhibitory control are reduced. In bipolar disorder, impulsivity and altered reward processing may increase susceptibility to binge-type eating and other dysregulated food behaviors [33,109].

Eating disorders are substantially more common among individuals with bipolar disorder than in the general population [101,104,110]. Meta-analytic evidence indicates clinically relevant rates of binge-eating disorder and bulimia nervosa in bipolar populations [101]. In a large clinical sample, eating-disorder comorbidity was particularly frequent among women and was associated with an earlier onset and a more severe bipolar course [110].

Binge eating frequently occurs during the evening or in response to prolonged daytime restriction, although eating timing has not been consistently reported in bipolar comorbidity studies. This omission limits the ability to distinguish primary chrono-nutritional disruption from eating-disorder psychopathology. Any assessment of time-restricted eating or fasting in bipolar disorder should therefore include formal screening for binge eating, compensatory restriction, and night eating.

### 6.8. Psychotropic Medication as a Modifier of Appetite and Timing

Psychotropic medications can substantially influence appetite, weight, insulin sensitivity, and food reward. However, most research has examined changes in body weight and total caloric intake rather than the timing of eating. This is an important limitation because medication-related hunger may emerge preferentially at particular times of day.

Sedating medications administered in the evening may increase opportunities for eating before sleep, while medications associated with daytime somnolence may delay awakening and the first caloric intake. Conversely, nausea, gastrointestinal discomfort, or reduced appetite may lead to meal omission. Medication schedules requiring administration with food can also impose or reinforce specific meal times.

These effects are likely to differ across antipsychotics, mood stabilizers, antidepressants, and combination regimens. Future studies should record medication type, dose, administration time, appetite changes, sleep effects, and temporal patterns of food intake. Without these data, it is difficult to separate illness-related chrono-nutritional disruption from treatment-related behavioral changes.

### 6.9. Eating Disorders as Amplifiers of Bipolar Instability

The coexistence of bipolar and eating disorders may create mutually reinforcing cycles involving mood symptoms, restriction, binge eating, sleep disruption, weight change, and medication non-adherence. Lifetime eating-disorder prevalence in bipolar samples varies widely, reflecting differences in diagnostic methods, population characteristics, and bipolar subtype [104].

Binge-eating disorder has been estimated to occur in approximately one in eight individuals with bipolar disorder in pooled analyses, while bulimia nervosa also shows clinically meaningful comorbidity [101]. In youth with bipolar disorder, lifetime eating-disorder diagnoses have likewise been reported at substantial rates [111].

This comorbidity is relevant to chrono-nutrition because restrictive eating, binge episodes, nocturnal intake, and compensatory fasting may all alter feeding-fasting rhythms. These behaviors should not be interpreted exclusively as circadian phenomena. A personalized chrono-nutritional approach must distinguish between rhythm irregularity arising from bipolar symptoms and patterns driven by an eating disorder.

### 6.10. The Self-Reinforcing Chrono-Affective Loop

The available evidence supports a bidirectional conceptual model rather than a simple linear pathway. Mood symptoms can disrupt sleep, appetite, motivation, reward processing, and daily structure; these changes can destabilize meal timing and fasting intervals. Irregular food intake may then worsen glucose variability, appetite signaling, sleep, and metabolic alignment, potentially feeding back into fatigue, cognitive dysfunction, and affective instability. A possible sequence may begin with emerging mood symptoms, which disrupt sleep and daily routines and, in turn, lead to irregular or delayed food intake. This may promote metabolic and circadian misalignment, further impairing energy regulation and sleep and ultimately contributing to additional mood destabilization.

This loop is biologically plausible but not yet established as causal in bipolar disorder. Most available studies are cross-sectional, and direct temporal evidence showing that changes in meal timing precede mood episodes is lacking. Repeated real-time assessment using ecological momentary assessment, digital food logs, actigraphy, continuous glucose monitoring, and mood tracking will be necessary to test this model prospectively.

The central clinical implication is that chrono-nutritional patterns should be treated as dynamic, mood-state-sensitive behaviors. Changes in eating time, meal regularity, nocturnal intake, or fasting duration may provide useful contextual information when interpreted together with sleep, activity, medication changes, and emerging affective symptoms. Whether these measures can serve as early warning markers or therapeutic targets remains to be determined.

## 7. Mechanistic Pathways Linking Chrono-Nutritional Disruption to Affective Instability

The pathways below are supported mainly by indirect human and translational/preclinical evidence. Bipolar-specific mediation and causal sequencing have not been demonstrated.

### 7.1. Internal Desynchronization and Clock-Gene Signaling

The most immediate consequence of irregular or biologically mistimed food intake is a potential loss of coordination between central and peripheral circadian oscillators. While the suprachiasmatic nucleus remains primarily synchronized by the light-dark cycle, feeding time can shift molecular rhythms in the liver, pancreas, adipose tissue, gastrointestinal tract, and skeletal muscle [27,66]. When meals repeatedly occur at inconsistent times or during the biological night, peripheral tissues may receive metabolic signals that conflict with central circadian timing [24].

At the molecular level, nutrient-sensitive pathways interact with the core circadian machinery through insulin, adenosine monophosphate-activated protein kinase, mechanistic target of rapamycin, sirtuins, glucocorticoids, and cellular redox signals [64,112]. These interactions allow metabolic state to influence the expression and phase of clock-controlled genes involved in glucose handling, lipid metabolism, mitochondrial function, and inflammatory regulation [112].

In bipolar disorder, the potential consequences of this internal desynchronization may be greater because circadian amplitude and rhythm stability are already vulnerable [4]. However, direct studies demonstrating that irregular meal timing alters clock-gene expression in bipolar disorder are not yet available. This pathway should therefore be considered biologically plausible but clinically unconfirmed.

### 7.2. Glucose Variability, Insulin Resistance, and Brain Energy Availability

Glucose tolerance and insulin sensitivity vary across the circadian cycle and are generally less favorable during the evening and biological night [23,59]. Late eating, circadian misalignment, and recurrent changes in meal timing may increase postprandial glucose exposure even when caloric intake and meal composition remain unchanged [24,68].

Repeated postprandial hyperglycemia and compensatory hyperinsulinemia may contribute over time to insulin resistance, oxidative stress, endothelial dysfunction, and low-grade inflammation [113]. Insulin also acts within the central nervous system, where it influences synaptic plasticity, cognition, reward processing, and monoaminergic signaling [114]. Consequently, impaired insulin action may affect not only peripheral metabolism but also neural systems relevant to mood regulation.

This mechanism is especially pertinent to bipolar disorder, in which insulin resistance has been associated with a more chronic illness course, rapid cycling, cognitive impairment, and poorer treatment response [50]. Psychiatric disorders may also contribute bidirectionally to the development and progression of type 2 diabetes through sleep disruption, stress, inflammatory activation, medication exposure, and unhealthy behavioral patterns.

The evidence does not yet establish that meal-timing-related glucose variability directly precipitates bipolar mood episodes. Nevertheless, recurrent metabolic instability could reduce the reliability of energy supply to the brain and interact with pre-existing mitochondrial vulnerability, sleep disturbance, and psychotropic medication effects.

### 7.3. Metabolic Inflexibility and Mitochondrial Dysfunction

Mitochondria integrate nutrient availability, circadian information, oxidative metabolism, calcium signaling, inflammatory responses, and cellular stress adaptation. Mitochondrial abnormalities in bipolar disorder include altered oxidative phosphorylation, adenosine triphosphate production, redox balance, calcium homeostasis, and mitochondrial gene expression [43,51].

Recent models propose that mitochondrial dysfunction may vary with illness state and contribute to differences in energy mobilization between mania, depression, and euthymia [51]. Mitochondria may also represent a point of convergence between the metabolic and inflammatory abnormalities observed in bipolar disorder [115].

Regular feeding–fasting cycles support the temporal alternation between nutrient storage, substrate oxidation, ketogenesis, autophagy, and cellular repair [65]. By contrast, extended eating windows and irregular feeding may repeatedly activate anabolic pathways and reduce the duration of fasting-related cellular processes [81]. Such disruption could impair metabolic flexibility, defined as the capacity to adapt fuel oxidation to changes in nutrient availability and energetic demand.

The concept of metabolic plasticity has recently been proposed as a framework for understanding the interaction between circadian and metabolic dysregulation in bipolar disorder [34]. From this perspective, chrono-nutritional disruption may further challenge an already vulnerable system by exposing mitochondria to inconsistent cycles of glucose, lipids, hormones, and cellular energy demand. This remains a hypothesis requiring direct assessment through metabolomics, mitochondrial biomarkers, continuous glucose monitoring, and mood-state tracking.

### 7.4. Hypothalamic–Pituitary–Adrenal Axis and Cortisol Rhythmicity

The hypothalamic–pituitary–adrenal axis is regulated by both central circadian signals and behavioral inputs, including sleep, stress, activity, and meal timing [45]. Cortisol influences glucose production, insulin sensitivity, appetite, adipose distribution, and food reward, while food intake can acutely modify cortisol secretion and peripheral metabolic responses [47].

Irregular sleep and late-night eating may flatten or shift the diurnal cortisol profile and increase the overlap between elevated glucocorticoid activity and nutrient intake [29,68]. This overlap may favor hyperglycemia, visceral fat accumulation, and impaired metabolic recovery during the biological night.

Altered cortisol secretion and stress reactivity have been repeatedly described in bipolar disorder, although findings vary according to polarity, illness stage, medication, and sampling methods [46]. Chrono-nutritional disruption could therefore interact with pre-existing hypothalamic–pituitary–adrenal axis abnormalities, particularly during periods of sleep loss, psychosocial stress, or emerging mood episodes.

The relationship is likely bidirectional. Increased cortisol may promote high-energy food preference and emotional eating, whereas irregular intake may further destabilize endocrine rhythms. This reciprocal mechanism may be especially relevant in patients whose eating behavior is driven by emotional distress or who misinterpret affective activation as hunger [14].

### 7.5. Immune-Inflammatory Activation

Immune signaling follows circadian rhythms, and the timing of food intake can influence cytokine production, leukocyte activity, gut permeability, and postprandial inflammatory responses [47,55]. Recurrent eating during the biological night may increase exposure to nutrients at a phase characterized by reduced metabolic tolerance, potentially intensifying oxidative and inflammatory signaling.

Bipolar disorder has been associated with abnormalities in C-reactive protein, interleukin-6, tumor necrosis factor-α, and other inflammatory mediators, although no single marker is sufficiently specific for diagnostic or therapeutic use [116]. Inflammation has also been linked with cognitive dysfunction and neurobiological abnormalities in bipolar disorder [117].

Chrono-nutritional disruption may contribute to this inflammatory burden indirectly through glucose variability, insulin resistance, adiposity, sleep disruption, and altered intestinal-barrier function. Mitochondrial dysfunction may further amplify inflammatory signaling through increased reactive oxygen species and impaired cellular stress responses [111].

These pathways are biologically interconnected, but direct evidence that correcting meal timing reduces inflammation or improves mood in bipolar disorder is currently lacking. Available findings from other metabolic and clinical populations should not be interpreted as proof of efficacy in bipolar illness.

### 7.6. Gut Microbiota Rhythmicity and Intestinal-Barrier Function

The gut microbiota exhibits daily oscillations in composition, localization, and metabolic activity. These rhythms are shaped by the host circadian system, feeding–fasting cycles, dietary composition, bile-acid secretion, gastrointestinal motility, immune activity, and sleep [118,119].

Irregular meal timing can alter the availability of intestinal substrates and disrupt microbial rhythmicity, while the microbiota can reciprocally influence host circadian and metabolic regulation through microbial metabolites, immune signaling, and interactions with intestinal epithelial clocks [119,120]. This reciprocal organization suggests that chrono-nutritional disruption may affect not only microbial composition but also the temporal pattern of microbial function.

Systematic reviews indicate that time-restricted eating and fasting can modify gut microbial composition, although human studies remain few and heterogeneous [121]. More recent human evidence suggests that time-restricted eating can reshape the intestinal microbiome and immune-cell profiles, but these findings have not been replicated in bipolar populations [122].

Gut-microbiota alterations have been reported in bipolar disorder, including differences in microbial diversity and the abundance of taxa involved in inflammation and metabolic regulation [123]. However, the evidence remains inconsistent because of small samples, medication exposure, geographic variation, diet, body mass index, smoking, and methodological heterogeneity.

Psychotropic medications may themselves modify the gut microbiota, creating additional difficulty in distinguishing illness-related from treatment-related effects [124]. Therefore, future chrono-nutritional studies in bipolar disorder should assess meal timing, dietary composition, medication, sleep, and microbial sampling time simultaneously.

### 7.7. Microbial Metabolites, Bile Acids, and Short-Chain Fatty Acids

Gut microorganisms generate metabolites capable of influencing metabolic, immune, endocrine, and neural pathways. Short-chain fatty acids participate in intestinal-barrier maintenance, immune regulation, glucose homeostasis, and peripheral clock signaling [125]. They should not, however, be described as direct entrainers of the suprachiasmatic nucleus; their principal effects are more plausibly mediated through peripheral metabolic and immune pathways.

Bile acids also display circadian variation and act as signaling molecules through receptors such as farnesoid X receptor and Takeda G-protein-coupled receptor 5 [47]. Their synthesis and enterohepatic circulation are influenced by meal timing, hepatic clock function, and gut microbial metabolism. Disruption of feeding rhythms may therefore alter bile-acid signaling and downstream pathways involved in glucose regulation, inflammation, and energy expenditure.

Evidence connecting these metabolite rhythms specifically with bipolar disorder remains preliminary. Metabolomic studies in bipolar disorder have nevertheless identified abnormalities across lipid, amino-acid, oxidative, and energy-related pathways [126]. Future studies integrating timed stool collection, plasma metabolomics, bile-acid profiling, and mood monitoring could determine whether microbial and metabolic rhythms differ according to polarity or meal regularity.

### 7.8. Tryptophan-Kynurenine and Monoaminergic Signaling

Tryptophan metabolism represents a potential link among diet, inflammation, gut microbial activity, circadian rhythms, and neurotransmission. Tryptophan can be used for serotonin and melatonin synthesis or diverted through the kynurenine pathway, producing metabolites with neuroactive, immunomodulatory, and oxidative properties [127].

A systematic review and meta-analysis found evidence of kynurenine-pathway dysregulation in major depressive and bipolar disorders, although findings varied according to diagnosis, mood state, sample characteristics, and analytical methods [127]. Inflammatory activation can increase indoleamine 2,3-dioxygenase activity and shift tryptophan away from serotonin synthesis toward kynurenine metabolism.

Meal timing may influence this pathway indirectly through insulin, cortisol, inflammation, microbial metabolism, and the temporal availability of dietary amino acids. The gut microbiota also generates indole derivatives from tryptophan, some of which influence intestinal-barrier integrity and immune regulation [128].

At present, there is no direct evidence that irregular meal timing produces clinically meaningful changes in kynurenine metabolism in bipolar disorder. This pathway remains mechanistically attractive but should be presented as a research hypothesis rather than an established mediator.

### 7.9. Appetite-Regulating Hormones and Food-Reward Circuits

Leptin, ghrelin, insulin, glucagon-like peptide-1, peptide YY, and other appetite-related hormones show circadian and meal-related variation [47]. Their secretion is influenced by sleep duration, meal composition, fasting intervals, adiposity, and metabolic status. These signals communicate with hypothalamic and mesolimbic circuits that regulate hunger, satiety, motivation, and food reward.

Meta-analytic evidence suggests abnormalities in selected appetite-regulating hormones in bipolar disorder, although these findings are strongly affected by body mass index, mood state, and medication [107]. Irregular eating may weaken anticipatory hormonal responses and increase the discrepancy between homeostatic hunger and reward-driven intake.

This mechanism may help explain why some patients alternate between prolonged meal omission and impulsive or emotional eating. Food intake may then become increasingly driven by dysphoria, reward seeking, or environmental cues rather than by coordinated feeding-fasting signals [14]. Nevertheless, prospective evidence linking hormone rhythms, eating time, and mood transitions in bipolar disorder is absent.

### 7.10. Oxidative Stress, Neuroplasticity, and Blood–Brain Barrier Integrity

Circadian misalignment, postprandial hyperglycemia, mitochondrial dysfunction, and inflammation can increase oxidative stress and impair cellular resilience [43]. Oxidative injury may affect membrane lipids, proteins, mitochondrial DNA, and signaling pathways involved in synaptic plasticity.

Brain-derived neurotrophic factor and other neuroplasticity-related signals vary according to sleep, activity, metabolic state, and inflammation. Although inflammatory and neurotrophic biomarkers have been investigated as potential indicators of treatment response in bipolar disorder, current evidence remains inconsistent [116].

Systemic inflammation and metabolic dysfunction may also affect blood–brain barrier permeability. A recent systematic review identified preliminary evidence of altered blood–brain barrier markers in bipolar disorder, but methodological heterogeneity prevented firm conclusions [129]. It is plausible that chronic chrono-metabolic disruption could contribute indirectly to barrier dysfunction through endothelial stress, inflammation, and oxidative damage, but this relationship has not been tested directly.

### 7.11. An Integrated Mechanistic Framework

The available evidence supports an interconnected rather than compartmentalized model. Irregular or biologically mistimed food intake may desynchronize peripheral clocks and impair glucose–insulin regulation. Recurrent metabolic stress may then reduce mitochondrial flexibility, alter cortisol and appetite signaling, promote inflammatory activation, and disrupt microbial rhythmicity. These changes may influence tryptophan metabolism, oxidative balance, neuroplasticity, and brain energy availability, thereby lowering resilience to affective destabilization.

In individuals with bipolar disorder, these processes may interact with pre-existing circadian vulnerability, psychotropic medication effects, sleep disturbance, metabolic comorbidity, and mood-state-dependent behavioral changes. Chrono-nutritional disruption would therefore function less as an isolated causal factor than as an amplifier within a vulnerable biological network.

Most components of this framework are supported by convergent evidence from circadian biology, metabolic medicine, and experimental models rather than by bipolar-specific intervention studies. The model should consequently be regarded as hypothesis-generating. Establishing causality will require longitudinal studies that measure meal timing, circadian phase, sleep, glucose dynamics, inflammatory markers, metabolomics, microbiota, and mood symptoms within the same participants.

**Figure 3:**
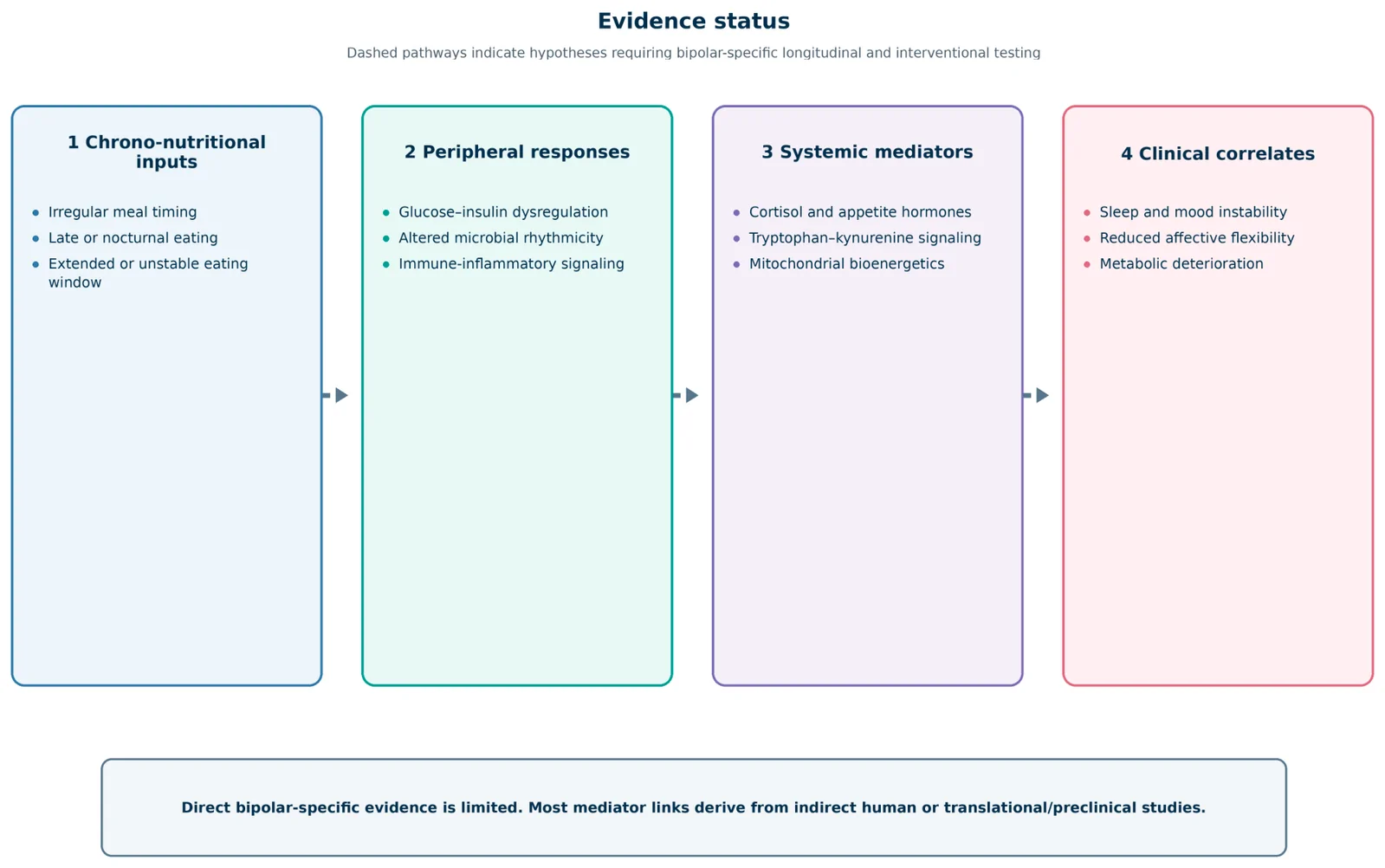
Evidence-coded molecular pathways potentially linking chrono-nutritional disruption to affective instability. Note. Direct bipolar-specific evidence for the complete pathway is limited; most mediator links are extrapolated from non-bipolar human studies or experimental models.

The proposed multi-level molecular pathways linking chrono-nutritional disruption to neurobiological and affective instability in bipolar disorder are summarized in Figure 3.

## 8. The Chrono-Nutritional Phenotype in Bipolar Disorder

This phenotype is provisional and is not a validated diagnostic, prognostic, or treatment-selection construct.

### 8.1. Conceptual Definition

A chrono-nutritional phenotype may be defined as the combination of circadian preference, sleep–wake organization, timing and regularity of food intake, daily eating-window duration, fasting intervals, and temporal distribution of energy intake. In bipolar disorder, this phenotype is unlikely to represent a single stable pattern. Rather, it may reflect the interaction among enduring circadian vulnerability, current mood state, psychotropic treatment, metabolic status, and environmental demands.

The available evidence does not yet support the identification of a distinct diagnostic chrono-nutritional phenotype specific to bipolar disorder. Several recurring characteristics, including eveningness, sleep irregularity, night eating, metabolic comorbidity, and disturbed daily routines, suggest the presence of clinically meaningful subgroups [17,39,130].

### 8.2. Eveningness and Delayed Behavioral Timing

The rvening chronotype is more frequent among individuals with bipolar disorder than among healthy controls and may persist independently of current mood state [39]. Eveningness has been associated with delayed sleep timing, poorer sleep quality, greater social jet lag, and more irregular behavioral rhythms [39,131].

Recent clinical evidence further suggests that eveningness in bipolar disorder may be associated with less healthy dietary habits, nocturnal eating, poorer quality of life, and a less favorable clinical course [54]. Nevertheless, evening chronotype should not be considered intrinsically pathological. Its clinical relevance depends on the degree of misalignment between individual circadian preference and externally imposed schedules.

From a chrono-nutritional perspective, eveningness may increase the probability of delayed first caloric intake, breakfast omission, later dinner, evening caloric concentration, and food intake close to sleep onset. However, these eating patterns should be measured directly rather than inferred from chronotype alone.

### 8.3. Sleep Irregularity as a Core Modifier

Subjective and objective sleep-timing disturbances are common in bipolar disorder [132]. Importantly, self-reported chronotype does not always correspond closely to objectively measured sleep timing, indicating that questionnaires and actigraphy capture related but non-identical dimensions [132].

Sleep irregularity may modify the chrono-nutritional phenotype through several pathways. Variable awakening times can alter the timing of the first meal, while delayed sleep onset and nocturnal awakenings can extend opportunities for evening or nighttime intake. Hypersomnia may compress daytime eating into a shorter interval, whereas insomnia and prolonged wakefulness may expand the daily eating window.

In euthymic patients, sleep disturbances remain common and may coexist with metabolic abnormalities [130]. This persistence suggests that chrono-nutritional assessment should not be limited to acute episodes but should also be performed during clinical remission.

### 8.4. Night Eating as the Best-Characterized Component

Night eating is currently the best-characterized chrono-nutritional disturbance in bipolar disorder. Studies have reported a higher prevalence of night eating syndrome among individuals with bipolar disorder than among healthy controls [93,94].

In euthymic bipolar I disorder, night-eating symptoms have been associated with eveningness, poorer sleep quality, and greater seasonality [94]. Sleep quality may partly mediate the relationship between chronotype and night eating, suggesting that delayed circadian preference does not operate independently of sleep disturbance [94].

Night eating may therefore identify a subgroup characterized by combined sleep, circadian, emotional, and metabolic vulnerability. However, current evidence is predominantly cross-sectional and cannot establish whether night eating precedes mood destabilization, emerges as a consequence of it, or reflects a shared underlying mechanism.

### 8.5. Metabolic Syndrome and Cardiometabolic Risk

Metabolic syndrome is common in bipolar disorder and may interact with circadian and sleep-related characteristics [130]. In a large cohort of euthymic patients, approximately one in five met criteria for metabolic syndrome, while more than half reported sleep disturbances and approximately one quarter showed an evening chronotype [130]. Evening chronotype was independently associated with higher triglyceride levels, whereas sleep disturbances were associated with greater abdominal circumference [130].

These findings do not prove that eveningness or irregular eating causes metabolic syndrome. Psychotropic medication, physical inactivity, smoking, diet quality, illness duration, socioeconomic conditions, and genetic predisposition remain important confounders. Nevertheless, they support the possibility that circadian phenotype contributes to heterogeneity in cardiometabolic risk.

A clinically useful chrono-nutritional phenotype should therefore include metabolic indicators such as body mass index, waist circumference, fasting glucose, glycated hemoglobin, insulin resistance, triglycerides, and blood pressure, rather than relying exclusively on eating questionnaires.

### 8.6. Mood Polarity and State-Dependent Variation

The chrono-nutritional phenotype may differ across manic, depressive, mixed, and euthymic states. Mania and hypomania may be associated with shortened sleep, prolonged nighttime activity, skipped meals, inconsistent caloric intake, and reduced adherence to daily routines. Bipolar depression may involve delayed awakening, breakfast omission, emotional eating, hyperphagia, reduced appetite, or increased evening intake.

These patterns should not be considered universal. Appetite direction, sleep duration, psychomotor activity, medication exposure, and comorbid eating psychopathology vary substantially among patients and across episodes. Consequently, chrono-nutritional characteristics observed during an acute episode should be distinguished from trait-like behaviors persisting during euthymia.

Repeated within-person assessment may be more informative than comparisons between patients and healthy controls. Changes from an individual’s usual meal timing, eating-window duration, or nocturnal intake may provide more clinically meaningful information than fixed population thresholds.

### 8.7. Rapid Cycling, Mixed Features, and Illness Severity

Circadian instability has been associated with clinically unfavorable characteristics of bipolar disorder, including greater episode burden, rapid cycling, suicidality, and impaired functioning [54]. Evidence linking these outcomes specifically to meal timing remains limited.

Patients with rapid cycling may experience repeated shifts in appetite, sleep, activity, and eating schedules as mood polarity changes. Mixed features may be associated with particularly unstable patterns because dysphoria, agitation, impulsivity, insomnia, and reward seeking can occur simultaneously.

At present, it cannot be concluded that irregular eating independently contributes to rapid cycling or mixed states. Prospective studies are needed to establish whether changes in meal regularity precede clinical deterioration or simply accompany emerging episodes.

### 8.8. Sex, Gender, Age, and Reproductive Stage

Chronotype and eating behavior vary according to age, sex, gender-related roles, occupational schedules, and reproductive stage. Younger individuals tend to show greater eveningness, whereas circadian timing generally advances with age [133]. This age effect may be clinically relevant because bipolar disorder often emerges during adolescence or early adulthood, when eveningness and social jet lag are already pronounced.

Women with bipolar disorder may experience greater rates of appetite and weight changes during depressive episodes and a higher prevalence of eating-disorder comorbidity [106,110]. Menstrual-cycle phase, pregnancy, the postpartum period, and menopause may further influence sleep, appetite, insulin sensitivity, and circadian timing, although direct chrono-nutritional evidence in bipolar disorder is scarce.

Gender-related caregiving, employment, household, and social responsibilities may also influence the feasibility and regularity of meal timing. Furthermore, the psychological and biological impact of major family transitions is not confined to female patients; emerging evidence highlights that depressive symptoms can significantly affect expecting fathers during the perinatal period as well, pointing to shared vulnerability pathways that cross gender boundaries during critical life events. Future studies should therefore distinguish biological sex from gender-related behavioral and social exposures rather than treating them as interchangeable variables.

### 8.9. Medication-Related Phenotypes

Psychotropic treatment may contribute to distinct chrono-nutritional phenotypes. Some antipsychotics and mood stabilizers increase appetite, weight, insulin resistance, and daytime sedation, while other medications may produce nausea, reduced appetite, activation, or sleep disruption.

The timing of medication administration may also influence eating behavior. Evening sedation may be accompanied by food intake shortly before sleep, while morning somnolence may delay awakening and the first daily meal. Medications requiring administration with food may impose a structured eating time, whereas complex regimens may reduce adherence to both meals and treatment.

Most pharmacological studies report weight and metabolic outcomes without assessing when appetite increases or food is consumed. Chrono-nutritional research should therefore record medication type, dose, administration time, duration, appetite effects, and temporal eating patterns.

### 8.10. A Provisional Clinical Phenotype

On the basis of current evidence, a provisional high-risk chrono-nutritional phenotype in bipolar disorder may include: evening chronotype or delayed circadian phase; irregular sleep–wake schedules; substantial day-to-day variability in meal timing; delayed first caloric intake; concentration of energy intake during the evening; nocturnal eating; extended or highly variable eating windows; poor alignment between meals, sleep, and medication schedules; metabolic syndrome or insulin resistance; comorbid emotional eating, binge eating, or night eating syndrome.

These characteristics should not be interpreted as diagnostic criteria or as a validated risk score. Several are supported only indirectly, and their relative contribution to mood instability remains unknown. The proposed phenotype is intended as a framework for structured clinical assessment and future hypothesis testing.

### 8.11. Trait, State, and Context

The chrono-nutritional phenotype of bipolar disorder can be understood across three interacting levels. The trait level includes chronotype, circadian vulnerability, metabolic predisposition, and habitual eating regularity. The state level includes episode-related changes in sleep, appetite, activity, and food intake. The contextual level includes employment, shift work, family schedules, food access, medication timing, travel, seasonality, and social obligations.

This distinction may help explain why the same patient can show different eating patterns across time and why apparently similar behaviors may have different clinical meanings. Late eating in a consistently delayed but stable individual differs from abrupt nocturnal eating emerging alongside reduced sleep and increased activation.

Accordingly, clinically meaningful assessment should focus not only on absolute timing but also on regularity, biological alignment, deviation from the individual’s baseline, and temporal association with mood symptoms. Such an approach may allow chrono-nutritional changes to be evaluated as potential markers of vulnerability, emerging destabilization, or treatment response.

## 9. A Proposed Chrono-Metabolic Model of Bipolar Destabilization

The model is explicitly hypothesis-generating and unvalidated. It organizes testable relationships rather than representing an established causal pathway.

### 9.1. Rationale for an Integrative Model

The available evidence suggests that chrono-nutritional disruption should not be considered an isolated dietary behavior. It may instead operate within a broader network connecting circadian vulnerability, metabolic regulation, sleep, stress responsivity, microbial activity, and mood stability. Bipolar disorder is characterized by disturbances in sleep–wake rhythms, social timing, energy regulation, and cardiometabolic health, while the timing of food intake influences many of the same physiological systems [4,17,34].

The proposed model therefore conceptualizes irregular or biologically mistimed food intake as a modifiable behavioral signal capable of amplifying pre-existing vulnerability. It does not assume that chrono-nutritional disruption is either necessary or sufficient to cause a mood episode. Rather, its effect is expected to depend on interactions with genetic predisposition, illness stage, mood polarity, sleep disruption, medication exposure, metabolic status, and environmental demands.

**Figure 4:**
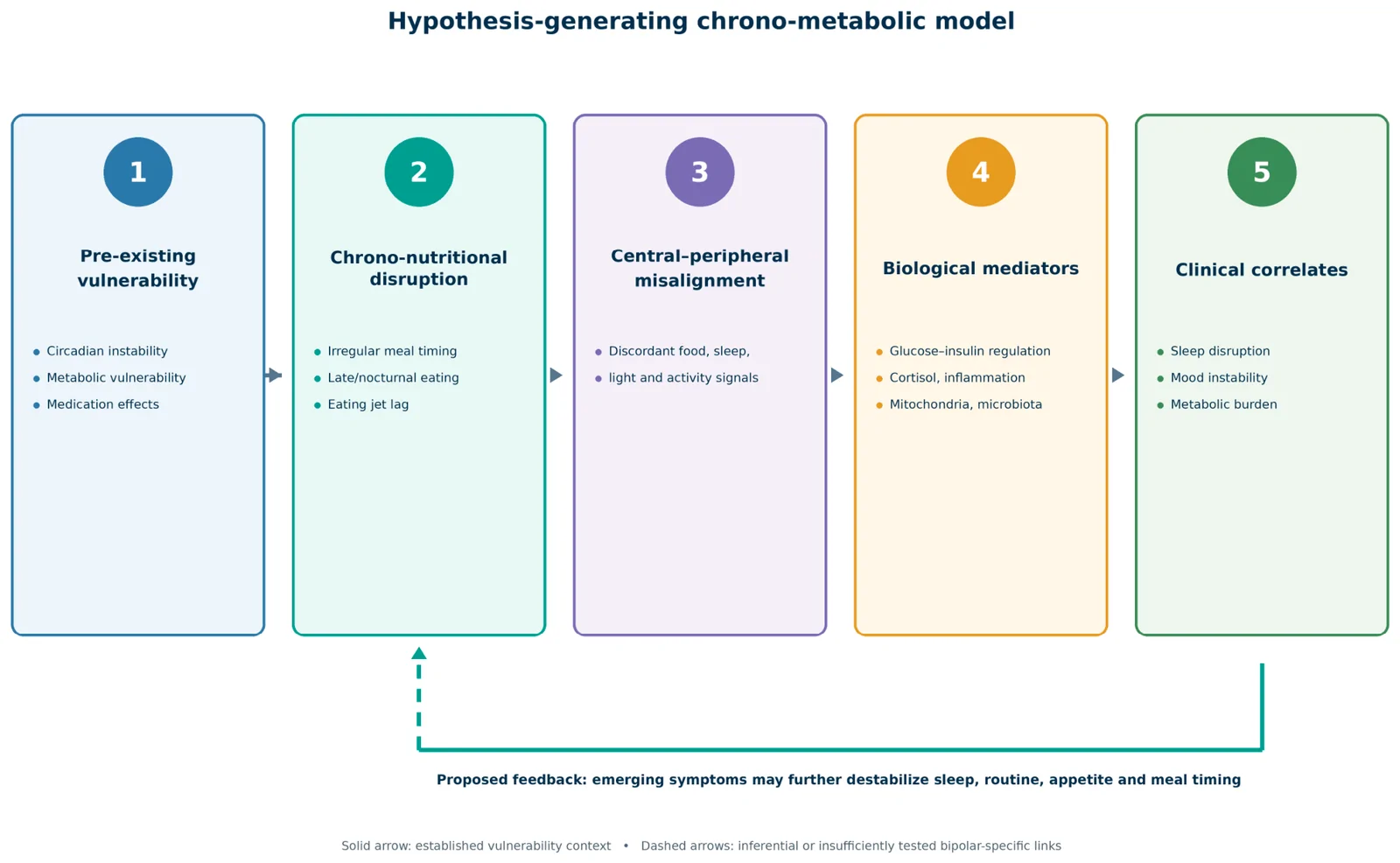
Integrated, hypothesis-generating chrono-metabolic model of bipolar destabilization. Note. Solid arrows denote relationships supported by established general circadian or metabolic evidence; they do not imply bipolar-specific causality. The dashed feedback arrow denotes a proposed bipolar-specific cycle. Direct bipolar evidence currently supports associations among night eating, eveningness, sleep disturbance, functioning, and metabolic correlates, whereas longitudinal and intervention evidence for meal timing remains limited.

The principal components and hypothesized bidirectional relationships of this integrative chrono-metabolic model are summarized in Figure 4.

### 9.2. Predisposing Circadian and Metabolic Vulnerability

The first component of the model is an underlying vulnerability involving reduced circadian stability and impaired adaptation to changes in environmental or behavioral timing. Individuals with bipolar disorder frequently show eveningness, variable sleep–wake schedules, altered melatonin and cortisol rhythms, social-rhythm disruption, and persistent circadian abnormalities during euthymia [4,39].

Metabolic vulnerability may coexist with this circadian phenotype. Insulin resistance, obesity, dyslipidemia, metabolic syndrome, mitochondrial dysfunction, and cardiovascular disease are overrepresented in bipolar disorder and may contribute to a more severe clinical course [50]. These abnormalities may reduce the capacity to accommodate recurrent changes in sleep, activity, food intake, and energetic demand.

From this perspective, circadian and metabolic vulnerability form the biological substrate upon which environmental and behavioral desynchronizers act.

### 9.3. Behavioral and Environmental Desynchronizers

Potential desynchronizing factors include sleep deprivation, delayed sleep timing, irregular social routines, shift work, transmeridian travel, nighttime light exposure, psychosocial stress, medication changes, and inconsistent physical activity [4,85,134].

Food-related desynchronizers may occur alongside these exposures and include irregular meal timing, delayed first caloric intake, late-night eating, nocturnal awakenings accompanied by food intake, extended eating windows, unplanned fasting, and substantial weekday-weekend variation. These behaviors may be induced or intensified by emerging mood symptoms, occupational schedules, emotional eating, medication-related appetite changes, or comorbid eating disorders [14,93,97].

The concurrence of several weak zeitgebers may be more destabilizing than any single behavior. For example, delayed sleep, irregular light exposure, late eating, and reduced daytime activity may jointly provide conflicting temporal information to central and peripheral oscillators.

### 9.4. Central-Peripheral Circadian Misalignment

Light primarily synchronizes the central pacemaker, whereas feeding–fasting cycles strongly influence clocks in peripheral metabolic tissues. When food intake repeatedly occurs at an inappropriate or inconsistent biological phase, peripheral oscillators may shift without a corresponding change in the central clock [27,66].

This internal desynchronization may impair the temporal coordination of glucose tolerance, insulin secretion, lipid metabolism, gastrointestinal activity, body temperature, cortisol secretion, and immune function [24,47]. Its magnitude may depend not only on clock time but also on chronotype, sleep phase, melatonin onset, and the regularity of the eating schedule.

In bipolar disorder, where sleep and social rhythms may already be unstable, irregular feeding could add a further source of temporal inconsistency. Direct evidence demonstrating central-peripheral misalignment caused by food timing in bipolar disorder is not yet available, and this component of the model remains inferential.

### 9.5. Metabolic and Neuroendocrine Mediators

Circadian misalignment and biologically late eating may impair glucose tolerance, increase postprandial glucose exposure, alter insulin secretion, and reduce the temporal efficiency of substrate utilization [23,71]. Extended eating windows may also shorten fasting intervals and weaken the separation between anabolic and catabolic processes [65].

These alterations may promote glucose variability, insulin resistance, and metabolic inflexibility. In the context of mitochondrial vulnerability, recurrent exposure to poorly timed nutrient signals may further impair energy production, redox balance, and adaptation to changes in fuel availability [43,51].

The hypothalamic–pituitary–adrenal axis provides an additional link. Sleep disruption, stress, and late eating may alter cortisol rhythmicity, while increased glucocorticoid activity can modify appetite, glucose metabolism, visceral adiposity, and food reward [46,47]. Chrono-nutritional disruption may therefore intensify a reciprocal interaction between stress-system activation and metabolic instability.

### 9.6. Inflammatory and Microbial Mediators

Metabolic stress, postprandial hyperglycemia, sleep disruption, and adiposity may promote low-grade immune-inflammatory activation. Inflammation may then impair insulin signaling, mitochondrial function, neuroplasticity, and circadian clock expression, creating a self-reinforcing biological loop [43,56].

Feeding rhythms also shape daily oscillations in the gut microbiota and its metabolic activity [118]. Irregular meal timing, nocturnal intake, sleep disruption, and medication exposure may alter microbial rhythmicity, intestinal-barrier function, bile-acid signaling, and the production of short-chain fatty acids and tryptophan-derived metabolites [19].

Microbial and inflammatory pathways should not be interpreted as independent causal chains. They interact with glucose regulation, appetite hormones, cortisol, mitochondrial function, and dietary composition. Moreover, current microbiota evidence in bipolar disorder remains limited by small samples, cross-sectional designs, medication effects, geographic variability, and inconsistent sampling procedures [19].

### 9.7. Reduced Energetic and Affective Flexibility

The convergence of circadian misalignment, glucose instability, mitochondrial stress, inflammation, altered appetite signaling, and microbial dysrhythmia may reduce metabolic flexibility. The organism may become less able to match energy production, substrate utilization, sleep, activity, and cognitive demand to changing environmental conditions [17,34].

This reduced energetic flexibility may have affective consequences. Fluctuating energy availability, impaired sleep, altered reward processing, fatigue, cognitive inefficiency, and stress sensitivity may lower resilience to mood destabilization. The clinical expression may differ according to pre-existing polarity: increased activation, sleep loss, and reward sensitivity could favor hypomanic or manic escalation, whereas fatigue, delayed timing, reduced motivation, and metabolic inefficiency could contribute to depressive persistence.

These state-specific pathways remain theoretical. The model does not imply that metabolic activation directly produces mania or that reduced energy availability alone produces depression. Mood episodes result from multiple interacting biological, psychological, and environmental processes.

### 9.8. The Self-Reinforcing Chrono-Affective Cycle

The model proposes a self-reinforcing cycle in which emerging mood symptoms disrupt sleep, activity, social routines, and food intake. These behavioral changes may promote further circadian and metabolic misalignment, impairing sleep quality, energy regulation, appetite signaling, and stress responsivity. The resulting biological instability may then contribute to worsening affective symptoms and further deterioration of daily routines.

For example, emerging hypomanic activation may reduce sleep and increase nighttime activity, leading to omitted daytime meals and irregular nocturnal intake. The resulting disturbance in glucose regulation, fasting duration, and peripheral-clock signaling may further impair sleep and behavioral control. Conversely, emerging bipolar depression may delay awakening and the first meal, reduce daytime activity, and concentrate food intake in the evening, potentially reinforcing delayed circadian timing and metabolic inefficiency.

This feedback structure may help explain why relatively small disruptions become clinically meaningful when repeated over several days in a vulnerable individual. However, prospective within-person evidence is required to determine whether chrono-nutritional changes precede, accompany, or follow mood-state transitions.

### 9.9. Protective Temporal Organization

The model also includes potentially protective factors. Stable sleep–wake schedules, regular meal timing, an adequate overnight fasting interval, avoidance of habitual nocturnal eating, consistent medication timing, daytime physical activity, and alignment between behavioral schedules and chronotype may strengthen temporal organization.

Interpersonal and social rhythm therapy provides indirect support for this principle because greater regularity of daily routines has been associated with improved clinical stability and a lower likelihood of recurrence [41]. The therapeutic contribution of meal timing cannot be separated from the broader stabilization of sleep, activity, and social rhythms in these studies.

Structured meal timing may therefore function best as one component of an integrated rhythm-stabilization strategy rather than as a stand-alone dietary prescription.

### 9.10. Clinical and Research Implications of the Model

The proposed model suggests that chrono-nutritional behaviors may serve as modifiable markers of biological and behavioral organization. Clinicians could assess recent changes in meal regularity, eating-window duration, first and last caloric intake, nocturnal eating, and alignment with sleep and medication schedules.

Abrupt deviation from an individual’s habitual eating rhythm may be more informative than adherence to a universal meal schedule. In some patients, increasingly irregular or nocturnal intake could accompany emerging activation, whereas delayed or compressed eating may parallel worsening depression.

Testing the model will require longitudinal studies combining repeated mood assessment; actigraphy and sleep monitoring; time-stamped dietary recording; continuous glucose monitoring; chronotype and circadian-phase assessment; medication timing; inflammatory and metabolic biomarkers; metabolomic and microbiome sampling.

These measures should be analyzed within individuals across mood states rather than exclusively through cross-sectional comparisons with healthy controls. Such designs could clarify whether chrono-nutritional disruption functions as a trait marker, a state marker, an early warning sign, a mediator, or merely a correlate of bipolar instability.

### 9.11. Scope and Limits of the Model

The chrono-metabolic model is intended as a conceptual framework rather than a validated causal account. Its individual components have different levels of evidential support. Circadian and sleep abnormalities, metabolic comorbidity, and social-rhythm disruption are well documented in bipolar disorder. Night eating and irregular eating behaviors have some direct clinical support, whereas the effects of meal timing on peripheral clocks, glucose metabolism, and microbial rhythms are derived primarily from experimental or non-bipolar populations.

The model should therefore not be used to attribute mood episodes to patients’ eating behavior or to recommend restrictive dietary interventions without appropriate clinical assessment. Instead, it provides a structure for integrating currently fragmented evidence and for designing bipolar-specific studies capable of testing the temporal relationships among food intake, metabolism, circadian rhythms, and mood.

## 10. Proposed, Non-Validated Assessment of Chrono-Nutritional Disruption in Bipolar Disorder

### 10.1. Rationale for a Multidimensional Assessment

Chrono-nutritional disruption cannot be adequately assessed through a single question concerning breakfast consumption or dinner time. Its clinical evaluation should integrate meal timing, eating regularity, sleep–wake organization, chronotype, mood state, medication timing, metabolic status, and comorbid eating psychopathology. These dimensions are interdependent, and the same eating behavior may have different implications according to its biological timing and clinical context [22,135].

Assessment should distinguish habitual characteristics from recent changes. A consistently delayed but stable eating schedule may reflect evening chronotype, whereas an abrupt shift toward meal omission or nocturnal intake may accompany emerging depression, hypomania, or mania. Within-person deviation from baseline may therefore be more clinically informative than comparison with a universal schedule.

At present, no validated bipolar-specific instrument comprehensively measures chrono-nutritional disruption. A pragmatic assessment should consequently combine standardized questionnaires, time-stamped dietary records, sleep and activity measures, clinical interviews, and selected metabolic indicators.

### 10.2. Chronotype, Social Jet Lag, and Sleep Timing

Chronotype can be assessed using the Munich Chronotype Questionnaire, which estimates habitual sleep timing separately on workdays and free days and permits calculation of corrected midsleep and social jet lag [136,137]. The reduced six-item µMCTQ may be useful when assessment burden must be minimized and has shown correspondence with the full questionnaire, actigraphy, and dim-light melatonin onset [138].

The Morningness–Eveningness Questionnaire is another widely used measure of circadian preference, although it assesses preferred timing rather than actual sleep behavior [139]. The two approaches are complementary: preference-based measures characterize subjective chronotype, whereas the Munich Chronotype Questionnaire captures habitual sleep timing under real-life social conditions.

Social jet lag is commonly estimated as the difference between midsleep on free days and workdays [78]. In bipolar disorder, this measure may identify conflict between endogenous timing and occupational or social schedules, but it should be interpreted together with employment status, shift work, caregiving responsibilities, insomnia, and hypersomnia.

A basic clinical assessment should document: habitual sleep onset and awakening on workdays and free days; variability in sleep timing; chronotype or morningness–eveningness; social jet lag; nocturnal awakenings; daytime sleep and napping; recent deviations from the patient’s usual sleep pattern.

### 10.3. Meal-Timing History

A structured meal-timing history should record the clock time and regularity of the first and last daily caloric intake, principal meals, snacks, nocturnal intake, and medication-associated eating. It should also assess the duration of the daily eating window and overnight fasting interval.

The Food Timing Questionnaire and Food Timing Screener were developed to assess food and sleep–wake timing and have demonstrated acceptable validity and reliability against established dietary and chronotype measures [140]. Although they have not been specifically validated in bipolar disorder, they may provide a more standardized approach than unstructured questioning.

Clinically relevant assessment should establish the usual timing of the first and last daily caloric intake and the extent to which these times vary across days. It should also determine whether meals are frequently skipped or substantially delayed, whether food is consumed after the intended sleep onset or during nocturnal awakenings, and whether eating schedules differ between workdays and free days. Additional attention should be paid to possible associations between evening or nighttime hunger and medication intake, as well as to recent changes in meal timing or appetite occurring in parallel with alterations in mood or sleep. The assessment should distinguish caloric intake from water, non-caloric beverages, and medication administration. It should also clarify whether breakfast omission is part of a stable eating pattern or results from delayed awakening, low appetite, disorganization, or late-night eating.

### 10.4. Food Diaries and Time-Stamped Dietary Recording

Prospective dietary records provide more accurate temporal information than retrospective estimates. A diary covering at least seven consecutive days, including both workdays and free days, can capture meal timing, day-to-day variability, eating-window duration, nocturnal intake, and weekday-weekend differences.

Each eating event should ideally include date and clock time; food and beverage consumed; approximate quantity; hunger and satiety; emotional state; social context; location; medication taken near the meal; sleep onset and awakening times.

Electronic records or smartphone applications may reduce recall bias by creating automatic time stamps. Photographic food records may further improve temporal accuracy, although they do not necessarily provide reliable estimates of energy or nutrient intake without additional analysis.

Dietary timing should be interpreted together with total energy intake and diet quality. A regular schedule based predominantly on nutritionally poor foods should not be considered metabolically protective solely because of its temporal consistency.

### 10.5. Ecological Momentary Assessment

Ecological momentary assessment allows repeated recording of mood, appetite, food intake, sleepiness, energy, stress, and impulsivity in the patient’s natural environment. This approach may be particularly valuable in bipolar disorder because retrospective reporting can obscure short-term fluctuations and temporal relationships among mood, behavior, and eating.

Repeated prompts can assess current mood and activation; hunger and food craving; recent food intake; emotional eating; impulsivity; fatigue and sleepiness; medication adherence; social context; perceived stress.

Ecological momentary methods can help determine whether eating episodes follow dysphoria, activation, sleep loss, or reward-related states. They may also reveal within-person patterns that are not apparent in conventional clinic visits. Adherence, response burden, reactivity to monitoring, and missing data must be considered [141].

Evidence on ecological momentary interventions in bipolar disorder remains preliminary, and these methods should currently be regarded primarily as research and monitoring tools rather than established therapeutic procedures [142].

### 10.6. Actigraphy and Wearable Devices

Actigraphy provides objective estimates of rest-activity rhythms, sleep timing, sleep duration, interdaily stability, intradaily variability, and activity amplitude. Systematic reviews indicate that bipolar disorder is associated with measurable alterations in rest–activity organization, although findings vary according to mood state, medication, recording duration, and analytic methods [143].

Prospective data suggest that greater variability in objective sleep timing may be associated with subsequent mood-episode relapse [144]. Earlier feasibility work also identified interdaily stability as a potentially relevant actigraphic parameter, although prediction accuracy was limited by small samples and missing data [145].

Wearable devices can complement food diaries by determining whether meals occur during the habitual active period, near sleep onset, or during nocturnal wakefulness. Consumer wearables may improve feasibility but differ in algorithmic transparency and measurement accuracy. Research-grade actigraphy remains preferable when precise circadian parameters are required.

### 10.7. Digital Phenotyping and Smartphone-Based Monitoring

Digital phenotyping combines active data, such as mood ratings and food logs, with passive data derived from smartphones or wearable devices. Passive measures may include mobility, geolocation, screen use, communication patterns, typing behavior, speech characteristics, activity, and sleep-related variables [146].

Systematic reviews suggest that portable digital technologies can detect behavioral differences among bipolar mood states and may support longitudinal symptom monitoring [147,148]. More recent studies using longitudinal wearable data and personalized machine-learning models have shown that individual behavioral patterns may help estimate mood symptom severity [149]. Nevertheless, methodological heterogeneity, small samples, algorithmic opacity, internal rather than external validation, and privacy concerns currently limit routine clinical implementation.

For chrono-nutritional research, digital phenotyping could integrate time-stamped eating events; sleep and activity; mobility and social behavior; mood self-ratings; medication timing; continuous glucose data; deviations from individual baseline patterns.

The objective should not be to infer food intake from digital behavior alone, but to examine whether changes in eating timing co-occur with alterations in sleep, activity, and mood.

### 10.8. Continuous Glucose Monitoring

Continuous glucose monitoring provides repeated interstitial glucose measurements and can characterize mean glucose, postprandial excursions, glucose variability, time in range, nocturnal glucose patterns, and responses to meals. It may be particularly useful in patients with obesity, prediabetes, type 2 diabetes, antipsychotic-associated metabolic risk, or suspected meal-timing-related glucose dysregulation.

Continuous glucose data should be synchronized with time-stamped meal records, sleep, activity, medication, and mood. Without these contextual data, glucose excursions cannot be attributed reliably to meal timing.

Automated detection of eating events from glucose signals is an emerging field, but current methods remain insufficiently accurate to replace dietary recording [150]. Continuous glucose monitoring should therefore be used as a complementary physiological measure rather than as a stand-alone measure of food intake.

In individuals without diabetes, the clinical meaning of short-term glucose variability is still uncertain. Its use in bipolar disorder is primarily investigational, and findings should not be overinterpreted as evidence of pathological dysglycemia.

### 10.9. Metabolic, Endocrine, and Circadian Biomarkers

Routine assessment should include established cardiometabolic indicators, particularly in patients receiving medications associated with weight gain or metabolic adverse effects. These may include body mass index and waist circumference; blood pressure; fasting glucose and glycated hemoglobin; fasting lipid profile; liver function; fasting insulin and indices of insulin resistance when clinically justified.

Research protocols may additionally assess cortisol rhythms, melatonin timing, appetite-regulating hormones, inflammatory markers, metabolomics, microbiota, and clock-gene expression. Dim-light melatonin onset remains a robust marker of circadian phase but requires controlled sampling conditions and is generally impractical for routine psychiatric care.

Biomarker sampling time must be standardized because cortisol, glucose, lipids, immune mediators, hormones, and microbial metabolites show diurnal variation. Failure to record collection time, fasting status, sleep timing, and recent food intake can introduce substantial measurement error.

### 10.10. Mood State and Psychiatric Characterization

Chrono-nutritional data should always be interpreted in relation to current mood state and recent symptom trajectory. Clinical assessment should document bipolar subtype; current polarity; depressive, manic, hypomanic, and mixed symptoms; sleep need and sleep duration; psychomotor activation or retardation; impulsivity and reward seeking; anxiety and stress; substance use; suicidality; recent medication changes.

Validated symptom scales may include the Young Mania Rating Scale, Montgomery–Åsberg Depression Rating Scale, Hamilton Depression Rating Scale, or other appropriate instruments. Repeated brief mood ratings may be particularly useful when combined with daily eating and sleep monitoring. The key question is not only whether a patient eats late or irregularly, but whether this pattern is habitual, medication-related, environmentally imposed, or temporally associated with emerging mood change.

### 10.11. Screening for Eating Disorders and Night Eating

Before recommending fasting, time-restricted eating, or substantial changes in meal timing, clinicians should assess for eating-disorder psychopathology. Screening should include binge eating, compensatory restriction, purging, body-image disturbance, loss of control over eating, nocturnal ingestion, and emotional eating [14,101].

The Night Eating Questionnaire may be used to assess night-eating symptoms, while structured diagnostic evaluation is required when night eating syndrome, binge-eating disorder, bulimia nervosa, or sleep-related eating disorder is suspected [91]. This distinction is essential because an extended eating window caused by insomnia differs clinically from recurrent binge eating, and nocturnal eating with awareness differs from parasomnia-related food consumption. Restrictive chrono-nutritional interventions may be inappropriate or harmful in patients with active eating disorders.

### 10.12. Medication and Substance-Use Assessment

Medication type, dose, administration time, sedation, activation, gastrointestinal effects, and appetite changes should be recorded. The assessment should determine whether food intake is required for medication administration and whether evening hunger follows sedating medication.

Alcohol, cannabis, stimulants, caffeine, nicotine, and other substances may also alter appetite, sleep, glucose regulation, and meal timing. Substance use should therefore be included in the chrono-nutritional history, particularly when nocturnal activity or irregular eating occurs.

Medication and substance-related effects should not be treated merely as confounders. They may be clinically meaningful contributors to the patient’s temporal eating pattern and should be incorporated into individualized management.

### 10.13. A Pragmatic Clinical Assessment Framework

A feasible clinical assessment could follow a stepped approach, with the intensity of evaluation tailored to the complexity of the individual case. At the first level, routine screening should be incorporated into the standard clinical interview and should explore chronotype, habitual sleep and wake timing, the timing of the first and last daily caloric intake, meal regularity, nocturnal eating, and any recent changes in appetite or eating schedules. The assessment should also consider the timing of psychotropic medication intake, particularly when medications influence appetite, sedation, or the need to consume food, as well as the presence of metabolic risk factors.

When chrono-nutritional disruption is suspected, a second level of structured monitoring may provide a more detailed characterization of daily patterns. Patients may be asked to complete a seven-day time-stamped food and sleep diary, ideally covering both working days and free days, in combination with a validated chronotype questionnaire, repeated mood ratings, and screening instruments for night eating, binge eating, or other disturbed eating behaviors. This approach may help identify day-to-day variability, delayed or prolonged eating windows, and temporal relationships among food intake, sleep, medication use, and mood fluctuations.

A third level of advanced phenotyping may be considered in research settings or in clinically complex cases in which routine assessment does not adequately clarify the relationship among circadian timing, eating behavior, metabolic regulation, and affective instability. Depending on feasibility and the specific clinical question, this level may include actigraphy, ecological momentary assessment, continuous glucose monitoring, assessment of dim-light melatonin onset, timed endocrine or inflammatory biomarkers, metabolomic profiling, and microbiome analysis. These measures may provide a more integrated characterization of central and peripheral circadian alignment, although their clinical utility in bipolar disorder remains to be established.

This stepped approach balances clinical feasibility with methodological precision. It also prevents unnecessary use of costly or burdensome measures in patients whose eating and sleep rhythms can be characterized adequately through routine assessment.

### 10.14. Interpretation and Current Limitations

No single threshold currently defines clinically significant chrono-nutritional disruption in bipolar disorder. Fixed cutoffs for “late” eating may be misleading because biological timing varies according to chronotype, sleep schedule, season, and social context.

Interpretation should therefore prioritize regularity across days; alignment with sleep and activity; distance between the last meal and sleep onset; nocturnal intake; deviation from the individual’s baseline; temporal association with mood symptoms metabolic and psychiatric context.

Digital tools offer opportunities for continuous, ecologically valid assessment, but they also raise concerns regarding privacy, data ownership, algorithmic bias, unequal access, patient burden, and clinical responsibility for responding to detected risk. Digital phenotyping remains promising but is not yet sufficiently standardized or externally validated for autonomous prediction of bipolar relapse [147,148].

The immediate clinical value of chrono-nutritional assessment lies in identifying modifiable disruption of daily organization and generating individualized hypotheses. Its use as a validated predictor of relapse or treatment response requires further prospective study.

## 11. Research-Oriented Therapeutic Implications

### 11.1. From Dietary Prescription to Temporal Organization

The therapeutic relevance of chrono-nutrition in bipolar disorder lies in shifting attention from dietary composition alone to the temporal organization of food intake. Nutritional quality remains essential, but the timing, regularity, and distribution of meals may provide an additional target within a broader strategy of circadian and metabolic stabilization [17,22].

At present, chrono-nutritional interventions should not replace established pharmacological, psychological, or lifestyle treatments for bipolar disorder. Their most appropriate role is as adjunctive strategies aimed at strengthening daily regularity, reducing nocturnal eating, improving metabolic organization, and supporting alignment among sleep, activity, medication, and food intake.

The immediate clinical priority is not necessarily to impose a restrictive eating window, but to reduce marked variability and restore predictable feeding–fasting rhythms. For many patients, a regular and sustainable schedule may be safer and more feasible than a rigid fasting protocol.

### 11.2. Stabilization of Meal Timing

Regular meal timing may provide peripheral metabolic systems with more predictable temporal signals and may reinforce the organization of the broader daily routine [22,74]. In bipolar disorder, this principle is consistent with the rationale of interpersonal and social rhythm therapy, which seeks to stabilize daily activities and reduce rhythm disruption [41].

For prospective research, an expert-informed regularization protocol could begin by testing whether relatively consistent times for the first and last caloric intake and principal meals are feasible and safe. The aim would be to reduce large day-to-day shifts without demanding rigid adherence; efficacy for bipolar outcomes remains unproven.

Regularity may be particularly relevant during euthymia and early recovery, when patients are more able to establish sustainable routines. During acute mania, severe depression, or mixed states, nutritional management may need to prioritize adequate intake, hydration, medication adherence, and safety rather than strict temporal optimization.

A pragmatic stepped framework for the assessment and management of chrono-nutritional disruption is presented in Table 2. The framework emphasizes individualized regularity and safety rather than universal meal schedules or prescriptive fasting.

### 11.3. Earlier Distribution of Daily Energy Intake

Human metabolic physiology generally favors nutrient intake during the earlier active phase, when glucose tolerance, insulin sensitivity, and diet-induced thermogenesis are more favorable [24,59]. Clinical studies and meta-analyses suggest that earlier meal timing or earlier time-restricted eating may produce modest improvements in body weight, waist circumference, glycemic control, and selected metabolic outcomes compared with later eating schedules [113,151].

In bipolar disorder, a gradual shift away from habitual late-night eating and excessive evening caloric concentration may therefore be metabolically reasonable. However, the recommendation should be individualized. A very early eating schedule may be poorly tolerated by patients with pronounced evening chronotype, delayed sleep phase, morning nausea, occupational constraints, or medications that affect appetite and alertness.

The therapeutic goal should be improved alignment rather than universal early eating. In some patients, advancing the final meal and reducing food intake close to sleep onset may be more feasible than substantially advancing the entire eating window.

### 11.4. Reduction in Nocturnal Eating

Nocturnal eating may overlap with insomnia, eveningness, emotional eating, night eating syndrome, medication-related hunger, and mood-state changes [93,94]. Treatment should therefore address its underlying mechanism rather than simply prohibiting food after a fixed hour.

When nocturnal eating is associated with insomnia or delayed sleep, interventions should include sleep and circadian management. When it follows emotional distress or loss of control, psychological treatment targeting emotional regulation and eating-disorder symptoms may be required [14]. When evening hunger emerges after medication administration, the pharmacological regimen, dosing time, and meal composition should be reviewed.

A gradual reduction in eating during the biological night may be appropriate, but abrupt restriction could worsen hunger, sleep, or binge-type behavior in vulnerable patients. Night eating syndrome and sleep-related eating disorder require specific diagnostic assessment and should not be managed through generic fasting advice.

### 11.5. Time-Restricted Eating

Time-restricted eating confines caloric intake to a consistent daily interval, usually without explicitly prescribing energy restriction or specific macronutrient targets [81]. Meta-analyses in non-psychiatric populations indicate modest benefits for body weight, waist circumference, fasting glucose, insulin, triglycerides, and other cardiometabolic outcomes, with earlier eating windows generally appearing more favorable than later windows [151,152].

Part of the observed benefit may result from reduced energy intake, less evening snacking, or improved behavioral regularity rather than from circadian realignment alone [82,152]. Moreover, the long-term superiority of time-restricted eating over conventional dietary counseling remains uncertain, and adherence varies according to work schedules, family routines, chronotype, and social context.

In bipolar disorder, time-restricted eating remains investigational. A randomized trial protocol has been developed to compare time-restricted eating with a Mediterranean dietary intervention in individuals with bipolar disorder and sleep or circadian disturbances [153]. A related pre-post protocol is examining emotional lability, quality of life, circadian phase, actigraphy, continuous glucose monitoring, clock-gene expression, and metabolic outcomes [153]. Until outcome data become available, time-restricted eating should not be presented as an evidence-based treatment for bipolar symptoms.

### 11.6. Personalized Rather than Rigid Eating Windows

Temporal regularization must not be conflated with rigid intermittent fasting. Eating windows shorter than 8 h may increase hunger, nutritional inadequacy, dehydration, medication-timing problems, and compensatory or binge-type eating. These risks may be amplified during mania or mixed states, when sleep loss, activation, impulsivity, and reduced attention to hydration are common, and during polypharmacy. Lithium requires stable hydration and sodium intake, while several atypical antipsychotics affect appetite and glucose regulation or require administration with food. Restrictive schedules should therefore be avoided during acute episodes and should remain confined to research or specialist care in stable, carefully screened patients. A broader, individually feasible and nutritionally adequate pattern of temporal regularity is conceptually distinct from fasting and may be studied without imposing a narrow eating window.

### 11.7. Integration with Interpersonal and Social Rhythm Therapy

Interpersonal and social rhythm therapy is based on the premise that disruption of daily routines can destabilize biological rhythms and contribute to bipolar recurrence [41]. Although meal timing has generally been included as one component of daily routine, its independent contribution has rarely been examined. Chrono-nutritional counseling could extend this framework by monitoring the regularity of first and last caloric intake, meal variability, nocturnal eating, and alignment with sleep and medication. The objective would be to integrate food timing into a broader rhythm-stabilization plan rather than treating it as an isolated dietary intervention.

This combined approach may be particularly appropriate because changes in eating time can serve as behavioral indicators of altered sleep, activation, motivation, or daily structure. Prospective studies should determine whether including meal-timing targets improves outcomes beyond conventional social-rhythm stabilization.

### 11.8. Integration with Sleep and Light Interventions

Food timing should be coordinated with sleep and light exposure because isolated modification of one zeitgeber may be insufficient when other behavioral signals remain misaligned. Advancing meals while maintaining late-night light exposure and irregular sleep may produce only partial alignment. A comprehensive chronotherapeutic strategy may include regular sleep and wake times appropriately timed morning light exposure; reduction in bright or blue-enriched light at night; structured daytime activity; regular meal timing limitation of habitual nocturnal eating; consistent medication administration.

Bright-light therapy and other chronotherapeutic interventions require careful clinical supervision in bipolar disorder because inappropriate timing or insufficient mood-stabilizer coverage may increase the risk of activation. Chrono-nutritional changes should similarly be introduced with attention to polarity and early signs of mood destabilization.

### 11.9. Integration with Pharmacotherapy

Psychotropic medication may influence appetite, sedation, glucose regulation, gastrointestinal function, and the timing of food intake. Chrono-nutritional interventions should therefore be coordinated with pharmacological treatment rather than prescribed independently. Clinical review should establish whether medication must be administered with food; produce evening hunger or daytime appetite suppression; cause morning sedation and delayed first intake; increase nausea or gastrointestinal discomfort; affect glucose regulation or body weight; have recently been initiated or dose-adjusted.

Meal timing should never compromise adherence or alter medication absorption without specialist guidance. In patients receiving lithium, particular attention should be given to stable hydration and sodium intake, while prolonged fasting or dehydration should be avoided. Patients taking glucose-lowering medication require additional monitoring because changes in meal timing may alter hypoglycemia risk.

### 11.10. Nutritional Quality Remains Essential

Temporal regularity cannot compensate for poor dietary quality. A chrono-nutritional intervention should preserve adequate energy intake, protein, fiber, micronutrients, and essential fatty acids while limiting excessive intake of ultra-processed foods, refined carbohydrates, and saturated fats.

The Mediterranean dietary pattern remains a reasonable framework because it emphasizes vegetables, fruits, whole grains, legumes, nuts, olive oil, and fish and has been widely studied in cardiometabolic and mental-health research [18]. In bipolar disorder, combining dietary quality with temporal regularity may be more appropriate than prioritizing either dimension alone.

This distinction is relevant to the ongoing comparison between time-restricted eating and a Mediterranean diet in bipolar disorder [153]. The two strategies target different dimensions: one primarily modifies timing, while the other primarily modifies food quality. Future interventions may need to integrate both.

Table 3 summarizes direct bipolar-specific studies most relevant to chrono-nutrition.

### 11.11. Patients Requiring Particular Caution

Restrictive or fasting-based interventions may be inappropriate or require specialist supervision in patients with current mania, hypomania, or mixed features severe bipolar depression with reduced intake or self-neglect; active anorexia nervosa, bulimia nervosa, binge-eating disorder, or other eating-disorder psychopathology; pregnancy or breastfeeding; childhood or adolescence; frailty or underweight; type 1 diabetes or diabetes treated with insulin or sulfonylureas; significant renal, hepatic, or gastrointestinal disease; substance-use disorders associated with irregular intake or dehydration; medications requiring food administration; a history of destabilization during fasting.

Religious fasting also requires individualized planning. The effects of altered sleep, nighttime eating, hydration, medication timing, and social rhythms should be considered together rather than focusing only on fasting duration.

Any chrono-nutritional intervention in bipolar disorder should include monitoring of both psychiatric and metabolic outcomes. Relevant psychiatric indicators include sleep duration, reduced need for sleep, activation, irritability, depressive symptoms, anxiety, impulsivity, suicidality, and adherence. Metabolic monitoring may include weight, waist circumference, glucose, glycated hemoglobin, lipids, blood pressure, and medication-related adverse effects.

The intervention should be reconsidered if it is associated with worsening sleep; increased activation or irritability; excessive hunger or binge eating; progressive dietary restriction; dizziness, dehydration, or hypoglycemic symptoms; impaired medication adherence; clinically significant weight loss; increased preoccupation with food or body weight.

Early therapeutic studies should incorporate predefined stopping criteria and adverse-event reporting rather than assuming that behavioral dietary interventions are inherently harmless.

### 11.12. A Stepped Clinical Approach

Role of nursing and mental-health professionals. In outpatient care, nurses may use brief, non-judgmental questions about first and last caloric intake, night eating, weekday–weekend shifts, sleep, hydration, and medication-with-food requirements, and may review a short time-stamped diary collaboratively. In inpatient care, meal refusal, nocturnal food seeking, abrupt changes from baseline, hydration, and the temporal relation between eating, sleep, medication, and activation can be documented during routine observations and communicated to the multidisciplinary team. Optional digital diaries or wearable data may help detect eating jet lag and social-rhythm changes, but alerts should be based on deviation from the individual baseline and require clinical interpretation. Monitoring must avoid weight-focused language, competitive fasting, rigid targets, or punitive feedback; signs of restriction, bingeing, food preoccupation, activation, reduced sleep need, or medication nonadherence require reassessment and, when indicated, dietetic or eating-disorder expertise. These procedures are proposed for feasibility testing and are not validated relapse-prevention interventions.

A practical chrono-nutritional strategy may be organized into three stages.

Stage 1, rhythm stabilization: the initial goal is to reduce major irregularity, establish predictable meal times, limit nocturnal eating, and coordinate food intake with sleep and medication.

Stage 2, metabolic optimization: once a stable routine is established, the timing of caloric distribution may be gradually shifted toward the earlier active period, while preserving dietary quality and adequate intake.

Stage 3, personalized time-restricted eating: a defined eating window may be considered only in selected, clinically stable patients without contraindications and with appropriate monitoring. At present, this stage should preferably occur within research protocols or specialist settings. This model prioritizes safety and sustainability. It also recognizes that regularity itself may be therapeutically meaningful even when a narrow eating window is neither feasible nor desirable.

The available evidence supports studying food-timing assessment within comprehensive management of sleep, routine, and metabolic health; it does not establish meal regularization or time-restricted eating as a mood-stabilizing treatment. Any practical suggestions in this section are expert-informed, non-validated proposals for prospective evaluation. Restrictive interventions should remain limited to research or specialist settings with explicit safety monitoring.

## 12. Research Gaps and Future Perspectives

The main limitation of the current literature is the scarcity of studies directly examining chrono-nutritional patterns in bipolar disorder. Most available hypotheses are extrapolated from circadian biology, obesity, diabetes, shift-work research, or other psychiatric populations and therefore cannot establish that meal timing has the same clinical relevance in bipolar disorder. Future studies should directly assess meal timing, day-to-day regularity, eating-window duration, nocturnal intake, fasting intervals, and daily caloric distribution, while also accounting for bipolar subtype, mood polarity, illness stage, psychotropic treatment, metabolic status, and comorbid eating disorders. Standardization is also urgently needed, as current definitions of breakfast skipping, late eating, night eating, eating jet lag, and time-restricted eating are highly heterogeneous. Chrono-nutritional variables should be interpreted relative to sleep timing, chronotype, social jet lag, and, when feasible, biological circadian phase rather than by clock time alone [22]. Longitudinal within-person designs are especially important to determine whether changes in eating timing precede mood destabilization, accompany prodromal symptoms, or arise only after an episode has begun. Repeated assessment combining mood ratings, actigraphy, time-stamped food records, medication timing, and metabolic measures could help distinguish stable trait-like patterns from state-dependent changes. Wearable and smartphone studies already suggest that rest–activity and circadian features may contribute to the detection of bipolar mood states, and the integration of food-timing data may improve these individualized models [154,155,156]. Randomized controlled trials are also required to compare meal regularization, reduction in nocturnal eating, earlier energy distribution, personalized time-restricted eating, and combined chrono-nutritional and social-rhythm interventions. These trials should include active comparators and evaluate psychiatric outcomes, metabolic outcomes, adherence, acceptability, and adverse effects. Particular attention should be given to safety in patients with mania, mixed features, severe depression with reduced intake, diabetes, eating disorders, pregnancy, frailty, or medications requiring food administration. Medication type, dose, timing, appetite effects, sedation, and metabolic impact should be incorporated prospectively rather than treated only as confounders. Objective and multimodal assessment using ecological momentary assessment, digital food logs, actigraphy, continuous glucose monitoring, timed endocrine sampling, metabolomics, and microbiome profiling may clarify the mechanisms linking food timing with affective instability. Biological sampling should be standardized according to clock time, circadian phase, fasting status, sleep, and recent food intake because many metabolic, inflammatory, hormonal, and microbial measures vary across the day. Gut-microbiota research should move beyond static taxonomic comparisons and examine microbial rhythmicity, timed metabolite production, intestinal-barrier function, and their relationships with meal timing and mood state [19]. Sex, gender, reproductive stage, age, shift work, socioeconomic conditions, food insecurity, cultural practices, and caregiving responsibilities should also be incorporated, as meal timing is shaped by both biology and social context. Ultimately, precision chrono-nutrition should not aim to impose a universal eating schedule, but to identify individualized patterns based on chronotype, sleep phase, mood polarity, metabolic phenotype, medication regimen, and eating-disorder risk. The most urgent priorities are to establish whether chrono-nutritional disruption predicts mood episodes, whether meal regularization improves outcomes independently of sleep and diet quality, and which patients can safely benefit from time-restricted eating. Until such evidence becomes available, chrono-nutrition should be regarded as a promising but still investigational component of personalized care in bipolar disorder.

## 13. Conclusions

Chrono-nutrition offers a potentially valuable framework for understanding how the temporal organization of food intake may interact with the circadian, metabolic, neuroendocrine, inflammatory, and microbial vulnerabilities associated with bipolar disorder. The current evidence suggests that irregular meal timing, delayed or nocturnal eating, unstable feeding-fasting cycles, and misalignment between food intake and sleep may contribute to internal desynchronization and impaired metabolic flexibility. However, direct bipolar-specific evidence remains limited, and most mechanistic pathways are supported by indirect clinical or experimental findings rather than by definitive longitudinal or interventional studies.

The available literature does not justify considering chrono-nutritional disruption an established causal factor for mood episodes or time-restricted eating a validated treatment for bipolar disorder. Nevertheless, food timing may represent a clinically relevant and modifiable component of broader rhythm instability. Its assessment may help identify changes in daily organization that accompany sleep disruption, emerging mood symptoms, medication-related appetite changes, or metabolic deterioration.

Current evidence supports only cautious clinical consideration derived from indirect evidence: clinicians may ask about meal timing when evaluating sleep, routine, medication adherence, eating-disorder risk, and cardiometabolic health. Regular and nutritionally adequate schedules may be reasonable adjunctive goals within established care, but they have not been demonstrated independently to improve bipolar outcomes. Restrictive interventions should remain investigational, individualized, and carefully monitored.

Future longitudinal and randomized studies should determine whether changes in meal timing precede affective destabilization, whether meal regularization provides benefits independent of sleep and diet quality, and which patients may safely benefit from personalized chrono-nutritional interventions. Until such evidence becomes available, chrono-nutrition should be regarded as a promising, hypothesis-generating, and potentially actionable dimension of precision care in bipolar disorder.

## Figures and Tables

**Table 1:** Clinically relevant forms of chrono-nutritional disruption in bipolar disorder.

Chrono-Nutritional Pattern	Operational Clinical Description	Potential Bipolar-Related Drivers	Proposed Biological Implications	Possible Clinical Significance	Current Evidence
Irregular meal timing	Marked day-to-day variability in the timing of meals or caloric intake	Unstable routines, mood fluctuations, sleep disruption, occupational demands	Unpredictable peripheral-clock entrainment; impaired coordination of metabolic responses	May accompany broader social-rhythm instability	Limited direct evidence; mainly indirect
Delayed first caloric intake	Long interval between awakening and first caloric intake	Eveningness, hypersomnia, depressive fatigue, reduced morning appetite, mania-related distractibility	Delayed metabolic activation; altered daily energy distribution	May indicate delayed circadian phase or emerging mood-related routine disruption	Indirect evidence
Breakfast skipping	Omission of the habitual morning meal	Delayed sleep, reduced self-care, prolonged nocturnal intake, intentional fasting	Effects depend on overall eating window, diet quality, and timing of the last meal	Not uniformly maladaptive; should be interpreted contextually	Mixed observational evidence
Late eating	Substantial caloric intake close to habitual sleep onset	Evening chronotype, insomnia, medication-related appetite, reduced daytime structure	Greater overlap with melatonin secretion; impaired glucose tolerance and substrate oxidation	Potential metabolic burden, particularly when inconsistent or biologically misaligned	Experimental metabolic evidence; limited bipolar-specific evidence
Nocturnal eating	Food intake during the habitual sleep period or after nocturnal awakenings	Insomnia, night eating syndrome, prolonged wakefulness, mood activation	Disruption of the fasting phase; adverse nocturnal glucose response	Associated with poorer sleep, anxiety, eveningness, and lower functioning in bipolar samples	Preliminary direct cross-sectional evidence
Extended eating window	Caloric intake distributed across most of the waking period or into the night	Frequent snacking, insomnia, emotional eating, medication-related hunger	Shortened fasting interval; reduced separation of anabolic and catabolic processes	May contribute to weight gain and metabolic inflexibility	Mostly indirect evidence
Unplanned or inconsistent fasting	Variable periods without food not resulting from a stable intervention	Mania, depression, impaired self-care, substance use, food insecurity	Variable glucose availability, dehydration risk, unstable feeding–fasting signaling	May interfere with medication intake and increase clinical instability	Clinical plausibility; insufficient direct evidence
Evening caloric concentration	Disproportionate percentage of daily energy consumed in the evening or night	Skipped daytime meals, delayed sleep, emotional eating, nocturnal activity	Less favorable postprandial metabolism and energy storage	Potentially relevant to obesity, insulin resistance, and sleep disruption	Indirect metabolic evidence
Eating jet lag	Substantial differences in meal timing between workdays and free days	Social schedules, shift work, weekend sleep delay	Repeated shifts in peripheral metabolic timing	May amplify social jet lag and rhythm instability	Emerging evidence; no established bipolar-specific prediction
Misalignment with medication	Meals do not correspond with medications that require food or consistent timing	Chaotic routines, appetite changes, sleep reversal	Variable absorption, tolerability, adherence, and metabolic effects	Clinically relevant for safety and treatment adherence	Clinically important but poorly studied

**Table 2:** Proposed, non-validated clinical assessment and management framework derived from the authors’ synthesis; items are intended for research and individualized clinical observation, not as evidence-based treatment guidance.

Clinical Domain	Suggested Assessment	Potential Warning Signs	Possible Clinical Response	Main Cautions
Meal timing and regularity	Timing of first and last caloric intake; meal variability across at least 7 days; weekday–weekend differences	Rapid changes from baseline; skipped meals; highly variable timing	Establish a predictable and nutritionally adequate meal schedule	Avoid rigid prescriptions during acute episodes
Eating window	Duration between first and last daily caloric intake	Very extended or highly variable window; recurrent night intake	Gradually consolidate intake into a sustainable daytime interval	Time-restricted eating remains investigational in bipolar disorder
Sleep–food alignment	Sleep diary, actigraphy when available, interval between final meal and sleep onset	Food intake during habitual sleep period; meals immediately before sleep; sleep reversal	Coordinate meal timing with sleep stabilization and morning light exposure	Consider chronotype rather than universal clock-time cutoffs
Mood state	Current polarity, mixed symptoms, activation, depressive slowing, appetite changes	Abrupt nocturnal eating, reduced intake during activation, compensatory evening eating	Prioritize stabilization of the mood episode and basic nutritional adequacy	Fasting interventions are inappropriate during mania or mixed states
Medication	Drugs requiring administration with food; appetite and weight effects; sedation timing	Missed doses, variable absorption, evening hyperphagia, medication-related hunger	Align meals with medication requirements; review regimen with prescribing clinician	Do not modify medication timing without clinical supervision
Metabolic health	Weight, waist circumference, blood pressure, fasting glucose or glycated hemoglobin, lipid profile	Insulin resistance, metabolic syndrome, rapid weight gain	Integrate dietary quality, physical activity, sleep, and metabolic monitoring	Meal timing alone is unlikely to correct established metabolic disease
Eating-disorder risk	Binge eating, night eating, restrictive eating, compensatory behaviors, body-image concerns	Active eating disorder, excessive dietary rigidity, fear of eating outside the window	Refer for specialist assessment; prioritize flexible and non-restrictive care	Structured fasting may worsen eating-disorder psychopathology
Social and occupational context	Shift work, caregiving, financial constraints, food insecurity, travel, cultural meal patterns	Schedules incompatible with stable meals; limited food access	Develop feasible individualized routines rather than idealized schedules	Avoid attributing socially determined disruption solely to patient behavior
Digital monitoring	Digital food logs, ecological momentary assessment, wearable-derived sleep/activity data	Increasing variability, deviation from personal baseline, reduced adherence	Use data collaboratively to identify patterns and support self-monitoring	Privacy, burden, unequal access, and false alerts
Advanced phenotyping	Actigraphy, continuous glucose monitoring, dim-light melatonin onset, timed biomarkers, microbiome or metabolomic analysis	Complex or research-relevant cases	Consider within specialized or research settings	Limited standardization and uncertain incremental clinical utility

Note. This conceptual framework is unvalidated. Regularization and monitoring of meal timing may be considered only as adjunctive elements of broader rhythm and metabolic care. Time-restricted eating is investigational in bipolar disorder.

**Table 3:** Direct bipolar-specific studies most relevant to chrono-nutrition.

Study	Design/Sample	Mood Phase	Exposure/Intervention	Main Outcome	Main Finding	Principal Limitations/Appraisal
Melo et al., 2018 [93]	Cross-sectional case–control; 80 bipolar patients, 40 controls	Euthymic	Night Eating Questionnaire	Night eating, sleep, anxiety, functioning	Night-eating symptoms were more prominent and clinically correlated in bipolar disorder.	Self-report; single center; cross-sectional; no temporal or causal inference.
Usta Sağlam et al., 2024 [94]	Cross-sectional bipolar sample	Not uniformly specified	Night eating, chronotype, seasonality	Sleep quality and night-eating symptoms	Night eating was associated with chronotype/seasonality through sleep-related pathways.	Cross-sectional mediation; self-report; residual confounding; no meal timestamps.
Godin et al., 2017 [130]	FACE-BD cross-sectional cohort; *n* = 752	Euthymic	Chronotype and sleep quality (not direct meal timing)	Metabolic-syndrome components	Evening chronotype was associated with triglycerides and atherogenic index.	Indirect proxy for food timing; cross-sectional; medication and lifestyle confounding.
Gershon et al., 2018 [132]	Cross-sectional multimethod study; limited sample	Clinically stable bipolar disorder	Subjective and objective chronotype	Agreement and clinical correlates	Objective and subjective eveningness were only partly concordant.	Not a food-timing study; limited generalizability; small sample.
Levenson et al., 2015 [134]	Prospective recurrence study; *n* = 118	Remitted after acute episode	Social-rhythm-disrupting events (meals not isolated)	Mood-episode recurrence	Greater rhythm disruption was associated with recurrence risk in partly adjusted models.	Meal timing not separable from other routines; residual confounding; association weakened with covariates.
Johnson et al., 2024 [153]	Randomized trial protocol; planned *n* = 200	Bipolar disorder with sleep/circadian disturbance	Time-restricted eating vs. Mediterranean diet	Mood symptoms, quality of life, sleep/circadian measures	Protocol establishes direct testing; no outcome data available at review cutoff.	Protocol only; efficacy and safety cannot yet be inferred.

Note. Chrono-nutritional patterns should not be interpreted in isolation or according to universal clock-time thresholds. Their clinical meaning depends on chronotype, sleep phase, mood state, medication regimen, total energy intake, diet quality, metabolic status, and deviation from the individual’s habitual pattern.

## Data Availability

No new data were created or analyzed in this study. Data sharing is not applicable to this article.

## Supplementary Materials

The following supporting information can be downloaded at: https://www.mdpi.com/article/10.3390/nu18183011/s1, Table S1: Database-Specific Search Strategies.
