# Supplementary material for: Chrono-Nutritional Disruption as a Potential Modifiable Factor in Bipolar Disorder
Source: Nutrients. 2026 Sep 14;18(18):3011. doi: 10.3390/nu18183011 (PMC13610716; doi:10.3390/nu18183011)
Supplement: Supplementary file 1 [file nutrients-18-03011-s001.zip › nutrients-4505874-supplementary.pdf]

Supplementary Table S1. Database-Specific Search Strategies

Search period: January 2000 to 30 June 2026. Earlier foundational studies were identified by backward citation searching as described in the manuscript.

Database	Fields/limits	Search strategy
PubMed/MEDLINE	Title/Abstract and MeSH where available; English; 2000–2026	("bipolar disorder"[Title/Abstract] OR "bipolar depression"[Title/Abstract] OR mania[Title/Abstract] OR hypomania[Title/Abstract] OR "mixed features"[Title/Abstract] OR "rapid cycling"[Title/Abstract] OR "mood instability"[Title/Abstract]) AND (chrononutrition[Title/Abstract] OR "chrono-nutrition"[Title/Abstract] OR "meal timing"[Title/Abstract] OR "meal regularity"[Title/Abstract] OR "irregular eating"[Title/Abstract] OR "breakfast skipping"[Title/Abstract] OR "late eating"[Title/Abstract] OR "night eating"[Title/Abstract] OR "feeding-fasting cycle"[Title/Abstract] OR "eating window"[Title/Abstract] OR "time-restricted eating"[Title/Abstract] OR "intermittent fasting"[Title/Abstract] OR "caloric distribution"[Title/Abstract]) AND ("circadian rhythm"[Title/Abstract] OR "circadian misalignment"[Title/Abstract] OR chronotype[Title/Abstract] OR "social jet lag"[Title/Abstract] OR metabolism[Title/Abstract] OR "insulin resistance"[Title/Abstract] OR inflammation[Title/Abstract] OR microbiota[Title/Abstract] OR "clock genes"[Title/Abstract])
Scopus	TITLE-ABS-KEY; article/review; English; 2000–2026	TITLE-ABS-KEY (bipolar disorder OR bipolar depression OR mania OR hypomania OR

		mixed features OR rapid cycling OR mood instability) AND (chrononutrition OR chrono-nutrition OR meal timing OR meal regularity OR irregular eating OR breakfast skipping OR late eating OR night eating OR feeding-fasting cycle OR eating window OR time-restricted eating OR intermittent fasting OR caloric distribution) AND (circadian rhythm OR circadian misalignment OR chronotype OR social jet lag OR metabolism OR insulin resistance OR inflammation OR microbiota OR clock genes)
Web of Science Core Collection	Topic (TS); article/review; English; 2000–2026	TS=(bipolar disorder OR bipolar depression OR mania OR hypomania OR mixed features OR rapid cycling OR mood instability) AND (chrononutrition OR chrono-nutrition OR meal timing OR meal regularity OR irregular eating OR breakfast skipping OR late eating OR night eating OR feeding-fasting cycle OR eating window OR time-restricted eating OR intermittent fasting OR caloric distribution) AND (circadian rhythm OR circadian misalignment OR chronotype OR social jet lag OR metabolism OR insulin resistance OR inflammation OR microbiota OR clock genes)

Supplementary mechanism-focused searches combined the bipolar-disorder block with each of the following: glucose-insulin regulation; mitochondrial bioenergetics; appetite-regulating hormones; hypothalamic-pituitary-adrenal axis; inflammatory signaling; tryptophan-kynurenine metabolism; gut microbial rhythmicity; and clock-gene expression. Syntax was adapted to each database.
